# Exploring the Luminescence, Redox, and Magnetic Properties in a Multivariate Metal–Organic Radical Framework

**DOI:** 10.1021/acs.chemmater.3c02460

**Published:** 2024-01-23

**Authors:** Gonçalo Valente, Pedro Ferreira, Miguel A. Hernández-Rodríguez, Carlos D. S. Brites, João S. Amaral, Pavel Zelenovskii, Filipe A. Almeida Paz, Samuel Guieu, João Rocha, Manuel Souto

**Affiliations:** † Department of Chemistry, CICECO-Aveiro Institute of Materials, University of Aveiro, Aveiro 3810-393, Portugal; ‡ Department of Physics, CICECO-Aveiro Institute of Materials, University of Aveiro, Aveiro 3810-393, Portugal; § Department of Chemistry, LAQV-REQUIMTE, University of Aveiro, Aveiro 3810-393, Portugal; ∥ CIQUS, Centro Singular de Investigación en Química Bioloxica e Materiais Moleculares, Departamento de Química-Física, Universidade de Santiago de Compostela, 15782 Santiago de Compostela, Spain

## Abstract

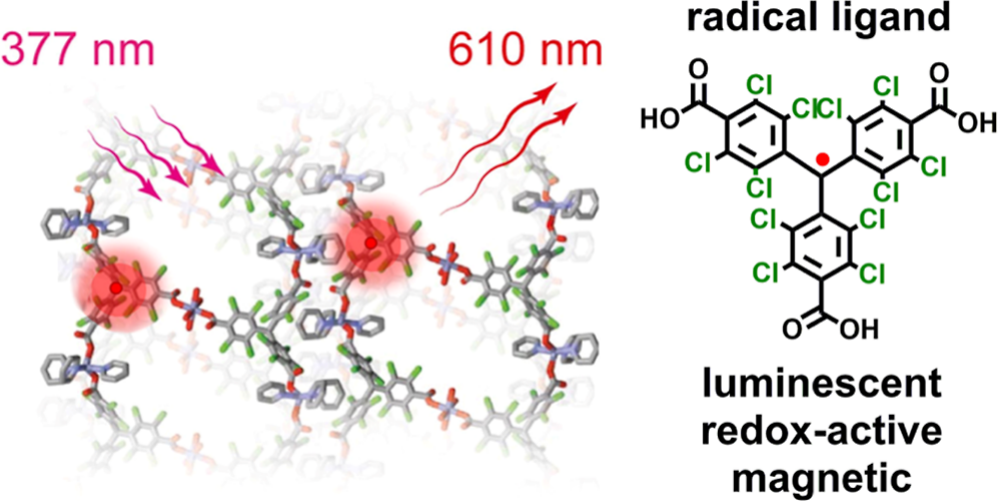

Persistent neutral organic radicals are excellent building blocks for the design of functional molecular materials due to their unique electronic, magnetic, and optical properties. Among them, triphenylmethyl radical derivatives have attracted a lot of interest as luminescent doublet emitters. Although neutral organic radicals have been underexplored as linkers for building metal–organic frameworks (MOFs), they hold great potential as organic elements that could introduce additional electronic properties within these frameworks. Herein, we report the synthesis and characterization of a novel multicomponent metal–organic radical framework (**PTMTC**^**R@NR**^**-Zn MORF**), which is constructed from the combination of luminescent perchlorotriphenylmethyl tricarboxylic acid radical (**PTMTC**^**R**^) and nonemissive nonradical (**PTMTC**^**NR**^) organic linkers and Zn(II) ions. The **PTMTC**^**R@NR**^**-Zn MORF** structure is layered with microporous one-dimensional channels embedded within these layers. Kelvin probe force microscopy further confirmed the presence of both organic nonradical and radical linkers in the framework. The luminescence properties of the **PTMTC**^**R**^ ligand (first studied in solution and in the solid state) were maintained in the radical-containing **PTMTC**^**R@NR**^**-Zn MORF** at room temperature as fluorescence solid-state quenching is suppressed thanks to the isolation of the luminescent radical linkers. In addition, magnetic and electrochemical properties were introduced to the framework due to the incorporation of the paramagnetic organic radical ligands. This work paves the way for the design of stimuli-responsive hybrid materials with tunable luminescence, electrochemical, and magnetic properties by the proper combination of closed- and open-shell organic linkers within the same framework.

## 1 Introduction

Persistent organic radicals are open-shell molecules with fascinating electronic, optical, and magnetic properties that have been explored as functional molecular materials toward a wide range of applications,^1,2^ including electronics,^3^ spintronics,^4^ energy storage,^5^ or biomedicine.^6^ Although most organic radicals are nonemissive, luminescent triphenylmethyl (trityl) radical derivatives with doublet electronic configuration have received a great deal of interest for applications in organic light-emitting diodes, circularly polarized luminescence, sensing, nanothermometry, etc.^7−14^ However, trityl radicals do not usually exhibit luminescence in the solid state due to quenching caused by exciton migration between nearby emissive radical centers.^10,11^ A recent strategy to solve this problem has been to prepare solid solutions with the corresponding nonradical derivatives to isolate the radical centers.^10^

Stable neutral π-radicals present a promising pathway for the construction of metal–organic frameworks (MOFs) and coordination polymers. Leveraging these radicals as building blocks provides an efficient strategy to imbue the resulting framework with enhanced electronic properties.^15,16^ While the use of neutral organic radical linkers in the creation of MOFs is not yet widespread, previous research study has utilized perchlorotriphenylmethyl (PTM) radical building blocks. These were used in the synthesis of porous coordination polymers specifically aimed at investigating the magnetic interactions between the paramagnetic linkers and transition metal ions.^16−18^ In particular, the perchlorotriphenylmethyl tricarboxylic acid radical (PTMTC) was combined with Cu(II) ions to obtain two-dimensional metal–organic radical frameworks (MORFs) that feature mesoporous hexagonal channels exhibiting a reversible expansion upon solvent adsorption, which in turn altered the magnetic ordering of the material.^17^ The same PTMTC ligand was also used for the synthesis of magnetic lanthanide-based MOFs in which the lanthanide luminescence was quenched by the PTMTC radical linkers.^19^ More recently, a 2D coordination polymer constructed from the tris(2,6-dichloro-4-pyridyl)methyl radical and Zn(II) ions has been reported. This material exhibited both luminescence^20^ and magnetoluminescence^21^ at low temperatures, thereby paving the way for the development of luminescent radical-based frameworks. However, the emission of this material rapidly decreased at a high temperature (∼250 K). In order to avoid solid-state fluorescence quenching,^22,23^ a promising strategy could be the synthesis of multivariate or multicomponent MOFs combining open- and closed-shell organic ligands within the same framework to isolate the luminescent radical species. Nevertheless, the construction of multivariate MOFs with radical and nonradical linkers remains unexplored.

Herein, we report the synthesis and characterization of a novel luminescent metal–organic radical framework (**PTMTC**^**R@NR**^**-Zn MORF**) based on the mixture of open- (**PTMTC**^**R**^**)** and closed-shell (**PTMTC**^**NR**^**)** perchlorotriphenylmethyl tricarboxylic acid linkers, which have a similar molecular structure but a different electronic structure, and Zn(II) ions. The intrinsic luminescence properties of the organic radical linkers, which were first studied in solution and in the solid state, are preserved within the framework due to the isolation of radical species. Redox and magnetic properties are also introduced into the framework from the radical linkers. The crystal structure of **PTMTC**^**R@NR**^**-Zn MORF** reveals the creation of a layered material featuring noninterpenetrated microporous one-dimensional channels within the layers. Upon exposure to ethanol, this material exhibits red luminescence and a reversible swelling behavior. This study demonstrates the possibility of modulating the luminescence, electrochemical, and magnetic properties of hybrid materials by doping with small traces of radical species in a controlled manner.

## 2 Results and Discussion

### 2.1 Synthesis and Characterization of **PTMTC**^**R**^

**Scheme 1:**
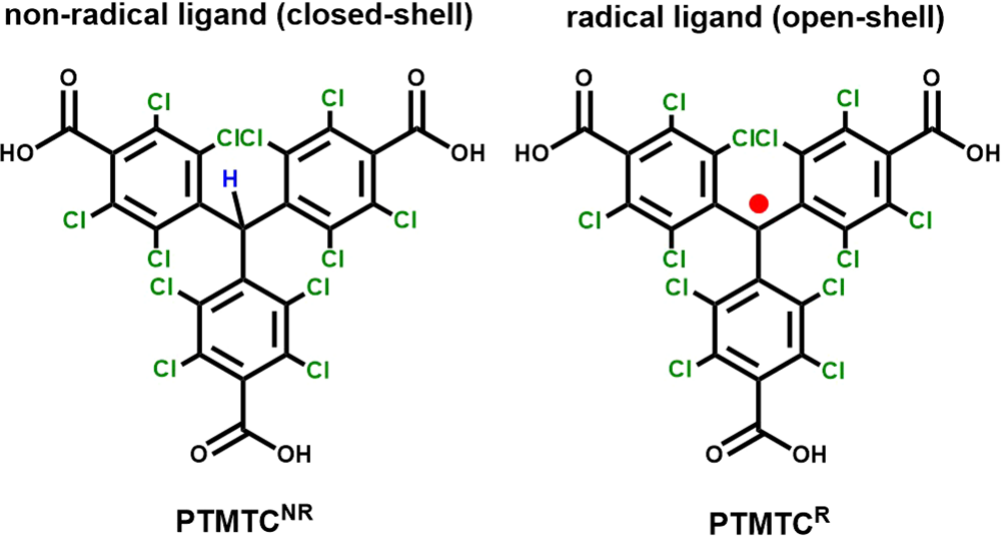
Molecular Structures of **PTMTC**^**NR**^ and **PTMTC**^**R**^

The perchlorotriphenyllmethyl tricarboxylic acid radical (**PTMTC**^**R**^) and nonradical (**PTMTC**^**NR**^) linkers (Scheme 1) were synthesized by optimizing a 4-step synthetic procedure to achieve high yields (Scheme S1).^24−26^**PTMTC**^**R**^ was characterized by IR (Figure S11), cyclic voltammetry (CV) (Figure S12), electron paramagnetic resonance (EPR) (Figure S13), magnetic susceptibility measurements (Figure S14), and mass spectrometry. CV of **PTMTC**^**R**^ shows a redox peak at −0.14 V (vs Ag/AgCl) assigned to the reduction of **PTMTC**^**R**^ to the anion state, whereas the EPR signal is consistent with the presence of organic radical species. The luminescence properties of **PTMTC**^**R**^ were initially studied in solution and in the solid state by preparing films of **PTMTC**^**R**^ on quartz substrates (see below).

### 2.2 Optical Properties of **PTMTC**^**R**^ in Solution and **PTMTC**^**R**^**@PTMTC**^**NR**^ Films

**Figure 1:**
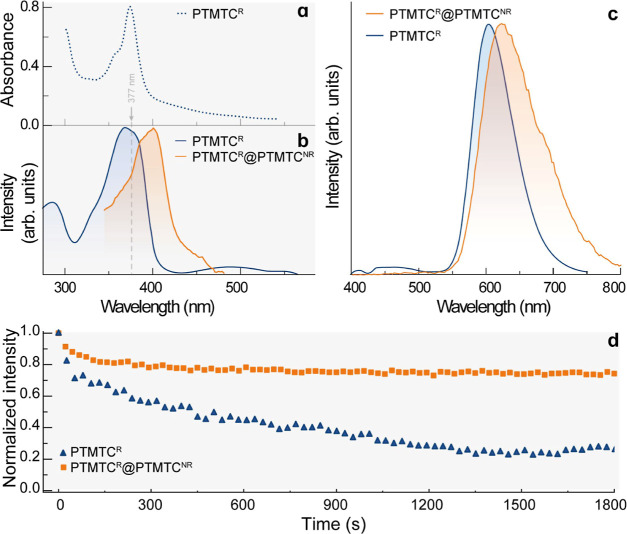
(a) Absorption spectrum of **PTMTC**^**R**^ in CHCl_3_ (0.05 mM). (b) Excitation monitoring at 607 nm and (c) emission upon 377 nm spectra of **PTMTC**^**R**^ in CHCl_3_ (0.05 mM) and **PTMTC**^**R**^**@PTMTC**^**NR**^ film deposited on the quartz substrate at room temperature. The arrow in (b) depicts the excitation wavelength used for recording the emission spectra presented in (c). (d) Time evolution of the emission intensity of **PTMTC**^**R**^ in CHCl_3_ (0.05 mM) and **PTMTC**^**R**^**@PTMTC**^**NR**^ film deposited on the quartz substrate over irradiation time by 378 nm excitation.

First, the optical properties of the **PTMTC**^**R**^ radical ligand in solution as well as those of thin films with a small concentration of radical species were studied for comparative purposes with the MOF luminescence properties. Note that the nonradical **PTMTC**^**NR**^ ligand is nonemissive in both solution and solid state. The absorption, emission, and excitation spectra of **PTMTC**^**R**^ (in CHCl_3_, 0.05 mM) and spin-coated **PTMTC**^**R**^**@PTMTC**^**NR**^ (2/8) films were measured at room temperature (Figure 1). The absorption spectrum of **PTMTC**^**R**^ (in CHCl_3_, 0.05 mM) in the UV–vis spectral range shows an intense absorption band near 378 nm, characteristic of the PTM radical chromophore,^27,28^ and a shoulder at 360 nm (Figure 1a). The excitation spectrum of **PTMTC**^**R**^ (in CHCl_3_, 0.05 mM) monitoring the 604 nm emission fits very well with the absorption spectrum, although the relative intensity of the peaks is dissimilar, the most effective route being the excitation at 377 nm (Figure 1b). The emission spectrum of **PTMTC**^**R**^ (in CHCl_3_, 0.05 mM) upon 377 nm irradiation shows a characteristic broad band centered at 605 nm with a full width at half-maximum of about 75 nm (Figure 1c). The emission spectrum of **PTMTC**^**R**^ recorded in a THF solvent (0.05 mM) presents a very similar emission band centered at 625 nm, suggesting a slight solvatochromic effect (Figure S15).

The photoluminescence quantum yield (PLQY) of **PTMTC**^**R**^ measured in CHCl_3_ (0.05 mM) is 6.1 ± 0.6% (upon 377 nm excitation), which is higher than the unsubstituted PTM (1.6 ± 0.2%).^8^ The solid **PTMTC**^**R**^**@PTMTC**^**NR**^ films were prepared by spin coating on quartz substrates in the dark using also different radical concentrations (wt%) (1, 2, 3 and 4%) to evaluate the influence of radical concentration on the PLQY. The PLQY of the films increased by decreasing the radical concentration in good accordance with the results reported elsewhere^10^ (see Table S1 and Figure S16), yielding to 3.2 ± 0.3% for a radical concentration of 1%.

Photostability studies were conducted on **PTMTC**^**R**^ to assess its suitability as a ligand in the crafting of luminescent MORFs. This was crucial because PTM radical derivatives can undergo ring-closure, leading to the formation of fluorenyl radical derivatives, particularly in solution.^29^ The photostability of **PTMTC**^**R**^ in a CHCl_3_ solution and deposited on a quartz substrate was evaluated upon continuous 378 nm irradiation at ∼1 mW·cm^–2^ (Figure 1d and Figures S17–S19). The **PTMTC**^**R**^**@PTMTC**^**NR**^ (2/8) film shows a maximum decrease of about 15%, whereas **PTMTC**^**R**^ dissolved in CHCl_3_ presents a decrease of about 60%. Moreover, the photobleaching dynamics of the film stabilizes after 200 s, whereas that of the CHCl_3_ solution stabilizes only after 1500 s. Given the promising photostability of the **PTMTC**^**R**^**@PTMTC**^**NR**^ films at low radical concentrations, we devised a similar approach for constructing the multivariate MORF.

### 2.3 Synthesis and Crystal Structure of **PTMTC**^**NR**^**-Zn** MOF and **PTMTC**^**R@NR**^**-Zn** MORF

Driven by the prospect of preparing hybrid coordination polymers incorporating isolated luminescent organic radical ligands, our aim was to synthesize multivariate or multicomponent MORFs combining open-shell **PTMTC**^**R**^ and closed-shell **PTMTC**^**NR**^ organic linkers. To avoid the quenching of the radicals luminescence, we chose Zn(II) as the metallic node due to its d^10^ closed-shell configuration.^30^ To use as a reference, we also synthesized the corresponding nonradical MOF using the closed-shell **PTMTC**^**NR**^ linkers. The slow diffusion of **PTMTC**^**NR**^ ligands and Zn(ClO_4_)_2_·6H_2_O in an ethanol/water mixture into a pyridine solution in ethanol yielded white needle-like crystals (**PTMTC**^**NR**^**-Zn MOF**) (Figure S20). Following the same procedure but using a mixture of **PTMTC**^**NR**^ and **PTMTC**^**R**^ with an 8/2 ratio (m/m), red crystals were obtained (**PTMTC**^**R@NR**^**-Zn MORF**) (Figure S21). These crystals were subsequently used to solve the crystal structures of **PTMTC**^**NR**^**-Zn MOF** and **PTMTC**^**R@NR**^**-Zn MORF**, which were also further characterized by IR (Figures S22–S23), EPR (Figure S24), magnetic susceptibility measurements (Figure 4a), and solid-state CV (Figures 4b and S25).

**Figure 2:**
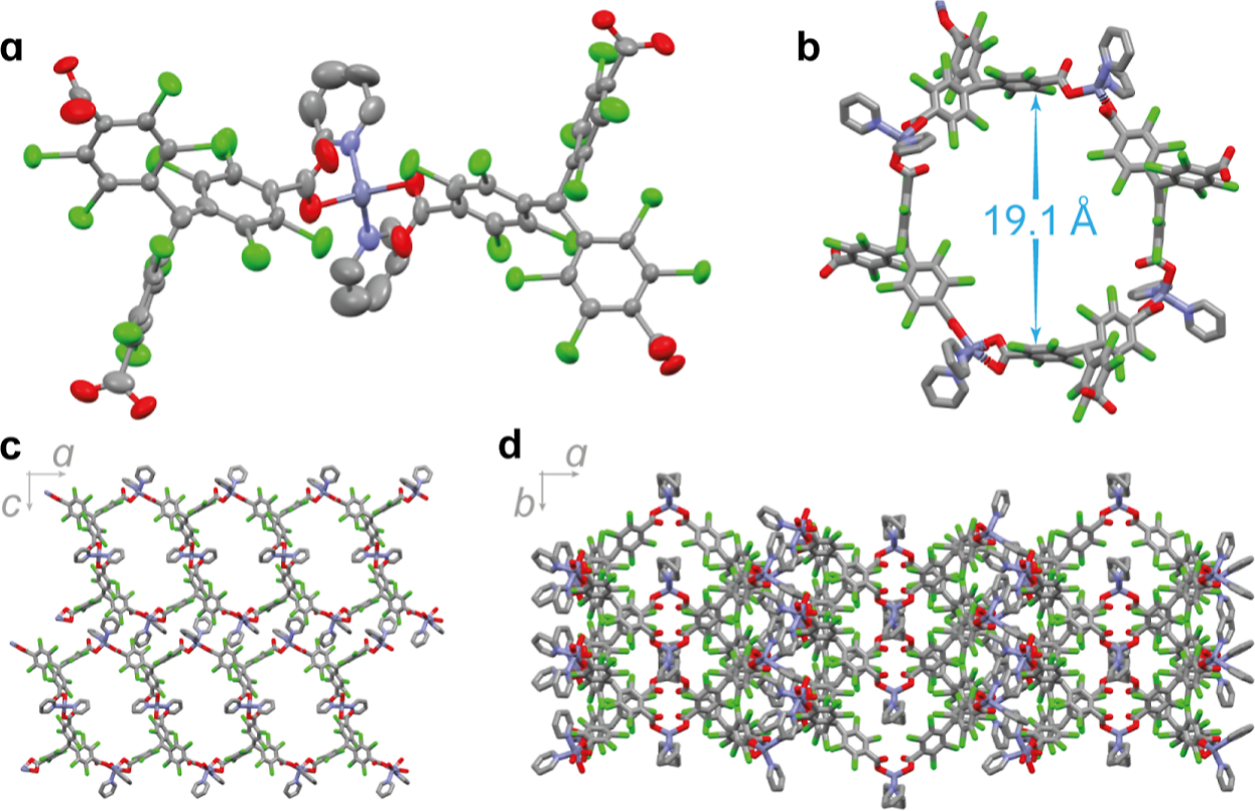
Partial views of the crystal structure of **PTMTC**^**NR**^**-Zn MOF** showing the (a) Zn(II) PTMTC^NR^-tricarboxylate building block, (b) microporous channel formed by four PTMTC^NR^ ligands and four Zn(II) metallic nodes, (c) arrangement of microporous channels along the *b*-axis, and (d) arrangement of the building blocks in the *ab* plane. Color code: C (gray), O (red), Cl (green), Zn (blue) atoms. For simplicity, solvent molecules and hydrogens are omitted.

Single-crystal X-ray diffraction data of **PTMTC**^**NR**^**-Zn MOF** and **PTMTC**^**R@NR**^**-Zn MORF** were collected by protecting the crystals with silicone grease to avoid solvent evaporation. It is important to highlight that both materials turn amorphous once the solvent evaporates, presenting a challenge in characterizing the desolvated material. **PTMTC**^**NR**^**-Zn MOF** crystallizes in the monoclinic space group *Cc,* and its crystal structure consists of tritopic **PTMTC**^**NR**^ linkers coordinated to octahedral Zn^2+^ units in a mono- or bidentate mode. In addition, pyridine and water molecules are also coordinated to the metal centers (Figure 2a). The structure exhibits microporous channels of ca. 19 Å × 19 Å along the *b*-axis (Figure 2b,c) formed by 4 **PTMTC**^**NR**^ linkers and 4 metallic nodes, with a total calculated solvent-accessible volume of ∼37% of the unit cell (Figure S26). Notably, the **PTMTC**^**NR**^**-Zn MOF** forms a layered structure along the *c*-axis while presenting a connected framework along the *b*-axis (Figure 2d). The average of the C–C distances between the central C atom of the PTM moieties and phenyl rings is ca. 1.55 Å, in accord with the reported distances for other nonradical PTM derivatives.^27,31^

**Figure 3:**
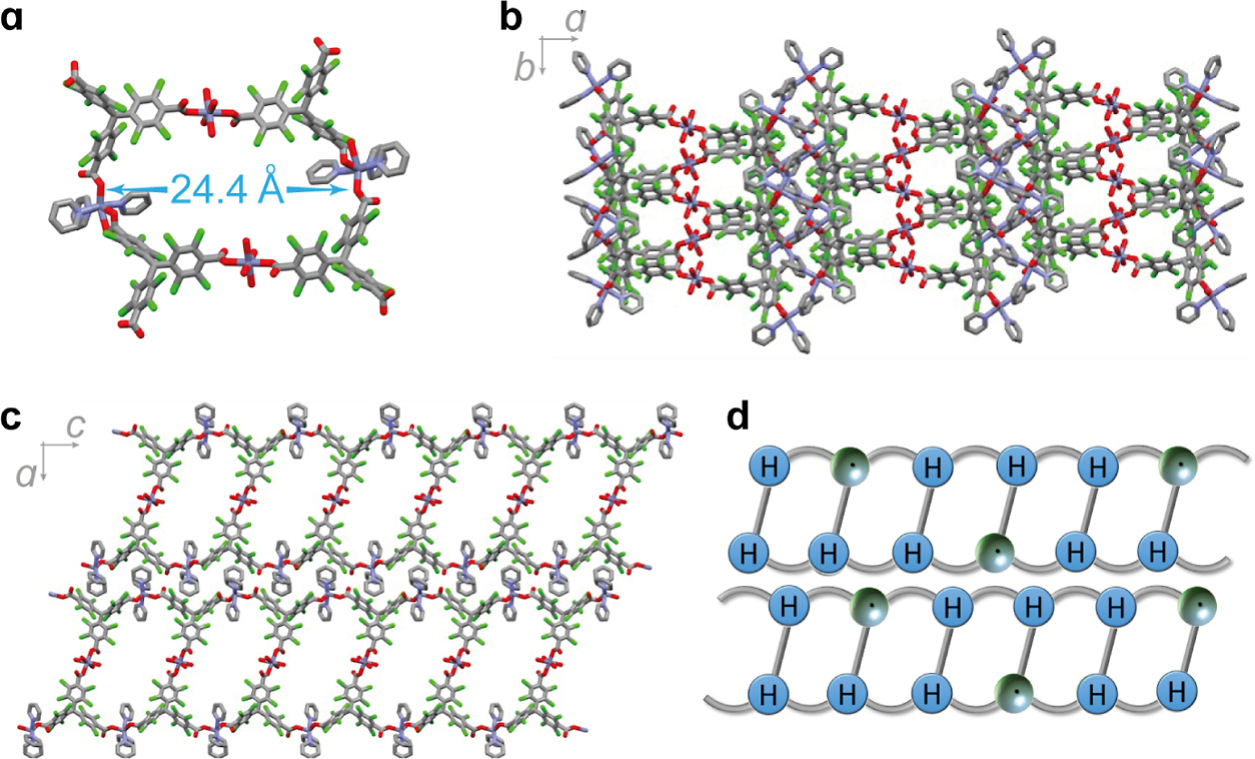
Partial views of the crystal structure of **PTMTC**^**R@NR**^**-Zn MORF** showing the (a) Zn(II) PTM-tricarboxylate building block, (b) arrangement on the *ab* plane, and (c) presence of microporous channels along the *b*-axis. Color code: C (gray), O (red), Cl (green), Zn (blue). For simplicity, solvent molecules and hydrogens are omitted. (d) Schematic representation of the 2D bilayer structures of **PTMTC**^**R@NR**^**-Zn MORF** on the *ac* plane with isolated radical moieties.

**PTMTC**^**R@NR**^**-Zn MORF** crystallizes in space group *P*2_1_/*c*, and the framework is based on the tritopic **PTMTC**^**R**^ and **PTMTC**^**NR**^ organic linkers and 6-fold Zn^2+^ ions with coordinated water or pyridine molecules as the metallic secondary building unit (SBU) (Figure 3a). Each Zn^2+^ unit is connected to two **PTMTC** organic linkers through mono- or bidentate carboxylate bonds forming infinite Zn-carboxylate chains along the *a*-axis (Figure 3b). Microporous noninterpenetrated 1D channels of ca. 15 Å × 24 Å are formed by 4 **PTMTC** moieties and 4 SBUs along the *b*-axis (Figure 3a,c), forming an open structure with a calculated free volume of ∼29% (see Figure S27) which is occupied by disordered ethanol and water solvent molecules. Such microporous channels are connected along the *c*-axis, but discontinuous along the *a*-axis, forming a layered structure as shown in Figure 3d. The structure is reminiscent (but not isostructural) of that of the PTMTC-Co(II) framework which was described as a (6,3)-helical network.^18^ Otherwise, the average C–C distance between the central *ipso*-carbon atom of the PTM unit and phenyl rings is slightly shorter (1.52 Å) than for **PTMTC**^**NR**^**-Zn MOF** (1.55 Å) but longer than that reported for PTM radical derivatives (1.46–1.48 Å).^27,31,32^ This intermediate value is consistent with having a mixture of radical and nonradical **PTMTC** organic ligands, as confirmed by the Kelvin probe force microscopy (KPFM) and magnetic susceptibility measurements (see below).

### 2.4 Kelvin Probe Force Microscopy

**Figure 4:**
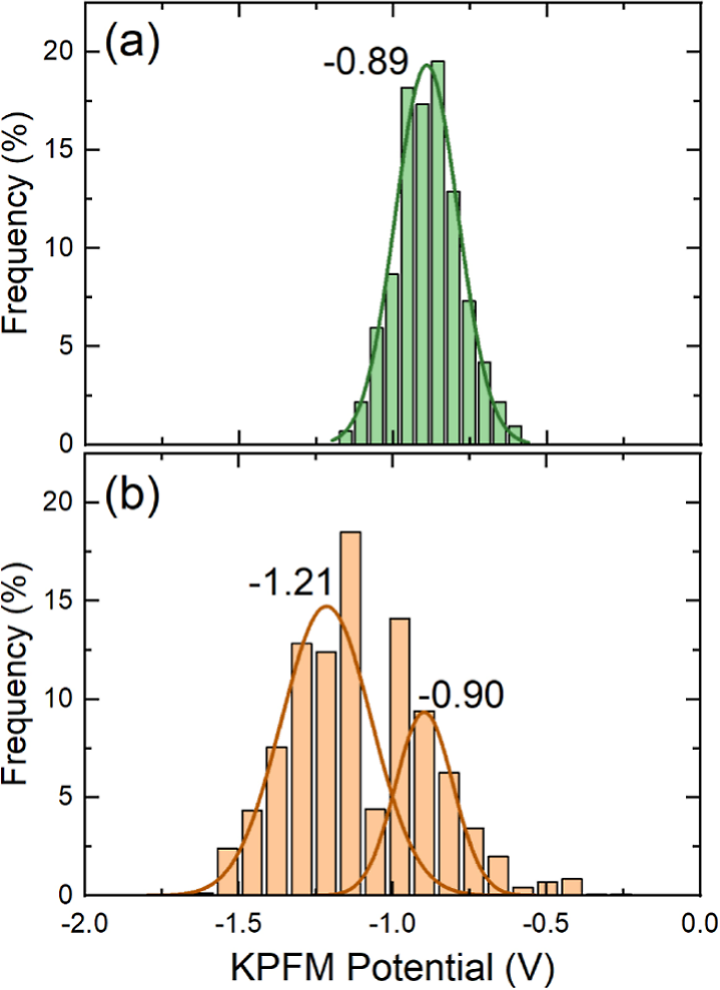
Distributions of KPFM potential over the surface of (a) **PTMTC**^**NR**^**-Zn MOF** and (b) **PTMTC**^**R@NR**^**-Zn MORF** crystals. Solid curves show the histogram fitting with the Gauss function.

KPFM measurements were performed on few **PTMTC**^**R@NR**^**-Zn MORF** crystals at room temperature (see SI for experimental details) to further confirm the presence of both organic radical and nonradical linkers within the framework. It has been reported that PTM nonradical and radical derivatives present different polarizabilities which may result in work function shifts.^33,34^ The sample’s work function (φ_s_) depends on the work function of the tip (φ_t_) and the KPFM amplitude, so *V*_KPFM_: φ_s_ = φ_t_ – *eV*_KPFM_, where *e* is an elementary charge. Therefore, for the same tip, the measured KPFM potential signal is proportional to the sample’s work function: *V*_KPFM_ = (φ_t_ – φ_s_)/*e*. The distribution of the KPFM potential at the surface of **PTMTC**^**NR**^**-Zn MOF** crystal represents a narrow unimodal distribution with the maximum at −0.89 V (Figure 4a and additional distributions in Figure S28) which was used as a reference. For **PTMTC**^**R@NR**^**-Zn MORF** crystals, the distribution consists of two peaks with characteristic maxima at −0.90 and −1.21 V (Figure 4b). The position of the first peak is close to that observed in the **PTMTC**^**NR**^**-Zn MOF** crystal.The distance between peaks positions (0.31 eV) is close to the shift of the work function between radical and nonradical PTM derivatives previously reported.^33,34^ Such a bimodal distribution of the KPFM potential is consistent with the presence of both nonradical and radical PTMTC linkers within **PTMTC**^**R@NR**^**-Zn MORF**. Note that KPFM measurements were only used as a qualitative technique to confirm the presence of both linkers, as these measurements were conducted on the surface of a limited number of crystals.

### 2.5 Magnetic and Electrochemical Properties of **PTMTC**^**R@NR**^**-Zn MORF**

**Figure 5:**
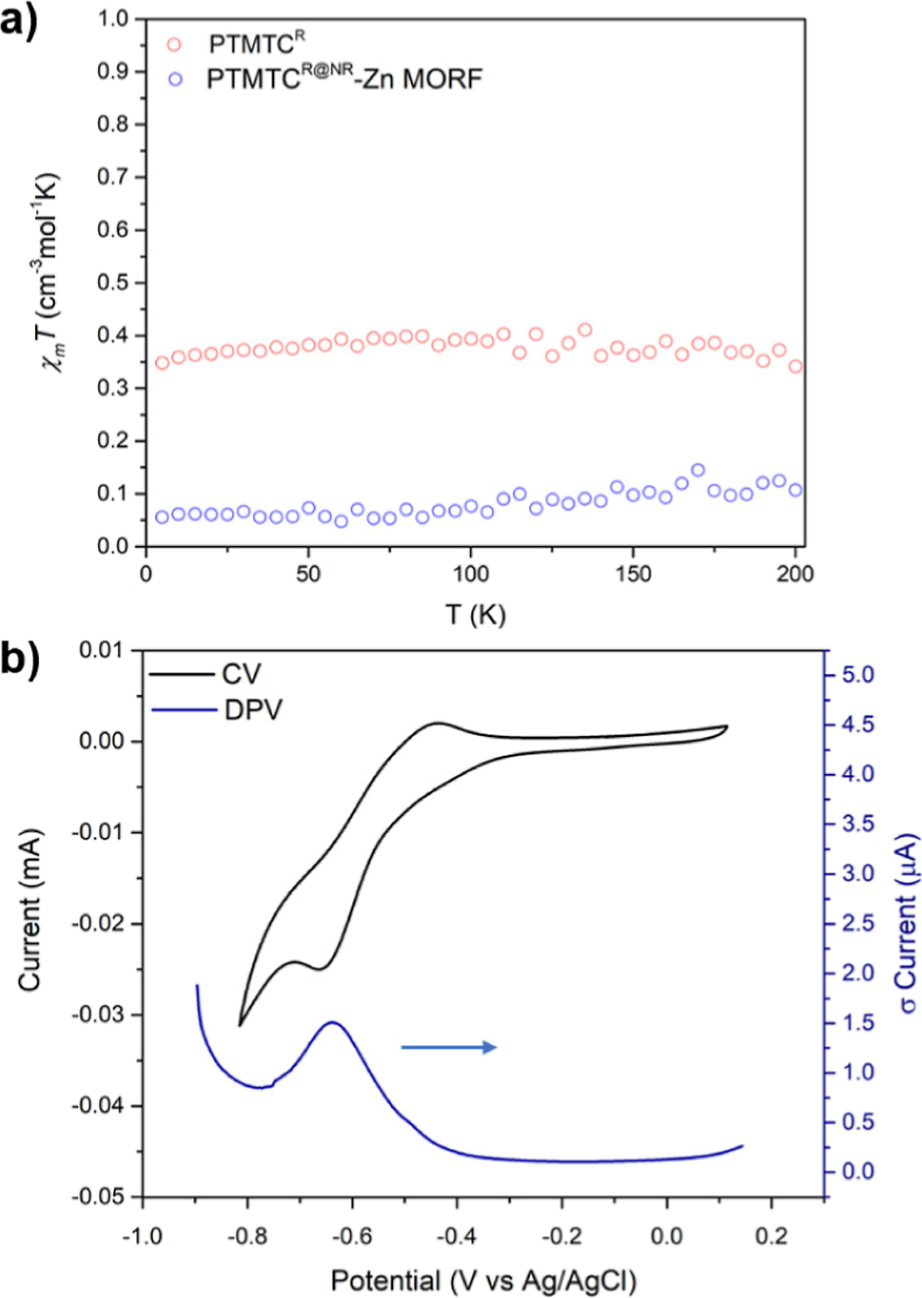
(a) Magnetic susceptibility χT versus temperature variation of **PTMTC**^**R**^ and **PTMTC**^**R@NR**^**-Zn MORF** in the 5–200 K range and at *H* = 5000 Oe. (b) Solid-state CV and DPV of **PTMTC**^**R@NR**^**-Zn MORF** in CH_2_Cl_2_ using TBAPF_6_ 0.1 M as the electrolyte and 0.3 V/s scan rate. Platinum wire was used as the counter electrode and silver wire as the pseudo reference electrode. Ferrocene was added as an internal standard. All potentials are reported versus Ag/AgCl.

The magnetic and electrochemical properties of **PTMTC**^**R@NR**^**-Zn MORF** were studied toestimate the percentage of radical linkers and confirm that the redox properties are maintained within the framework. The temperature dependence (5–200 K) of the magnetic susceptibility (χ) of **PTMTC**^**R**^ radical ligand showed a Curie–Weiss behavior (Figures 5a and S14) with a Curie constant of *C* = 0.35 cm^3^ K mol^–1^ and a χ_m_*T* close to the theoretical value (0.375 cm^3^ K mol^–1^), as expected for noninteracting *S* = 1/2 systems.^35^ On the other hand, the magnetic susceptibility of **PTMTC**^**R@NR**^**-Zn MORF** shows a lower χ_m_*T* value, which corresponds to ca. 15% of the theoretical value for two **PTMTC**^**R**^ noninteracting *S* = 1/2 spins (0.75 cm^3^ K mol^–1^) [the formula used was Zn_3_(PTMTC)_2_(py)_8_(H_2_O)_6_]. The EPR spectrum of a few single crystals of **PTMTC**^**R@NR**^**-Zn MORF** (Figure S24) was also consistent with the presence of a paramagnetic species. The number of paramagnetic species within the framework could be easily tuned, for example, by modifying the **PTMTC**^**R**^/**PTMTC**^**NR**^ ratio in the MORF synthesis. To evaluate the electrochemical properties of **PTMTC**^**R@NR**^**-Zn MORF** and further confirm the presence of radical species within the framework, solid-state CV and differential pulse voltammetry (DPV) were performed in CH_2_Cl_2_ using *n*-Bu_4_NPF_6_ as electrolyte at room temperature (Figures 5b and S25). The quasi-reversible redox process at *E*_red_^1/2^ = −0.55 V vs Ag/AgCl was assigned to the reduction of the electroactive PTMTC radical linkers to the anion species, appearing at more negative potentials than the **PTMTC**^**R**^ radical ligand in solution (*E*_red_^1/2^ = −0.14 V vs Ag/AgCl). This suggests that PTMTC radical moieties are more difficult to reduce when embedded in the framework. The peak-to-peak separation increases with the scan rate, whereas the linear relation between the cathodic and anodic peak current and the square root of the scan rate (Figure S25) suggests that the electrochemical process is diffusion controlled, which could be related to the poor diffusion of the electrolyte or partial dissolution of the ligand.^30,36,37^ The fact that the redox properties of the ligand are maintained in the framework opens new avenues for the design of electroactive radical-based MOFs as some PTM derivatives have been already explored for energy storage^38^ or electrocatalysis^39^, among other applications.

### 2.6 Solvent-Induced Structural Changes of **PTMTC**^**R@NR**^**-Zn MORF**

**Figure 6:**
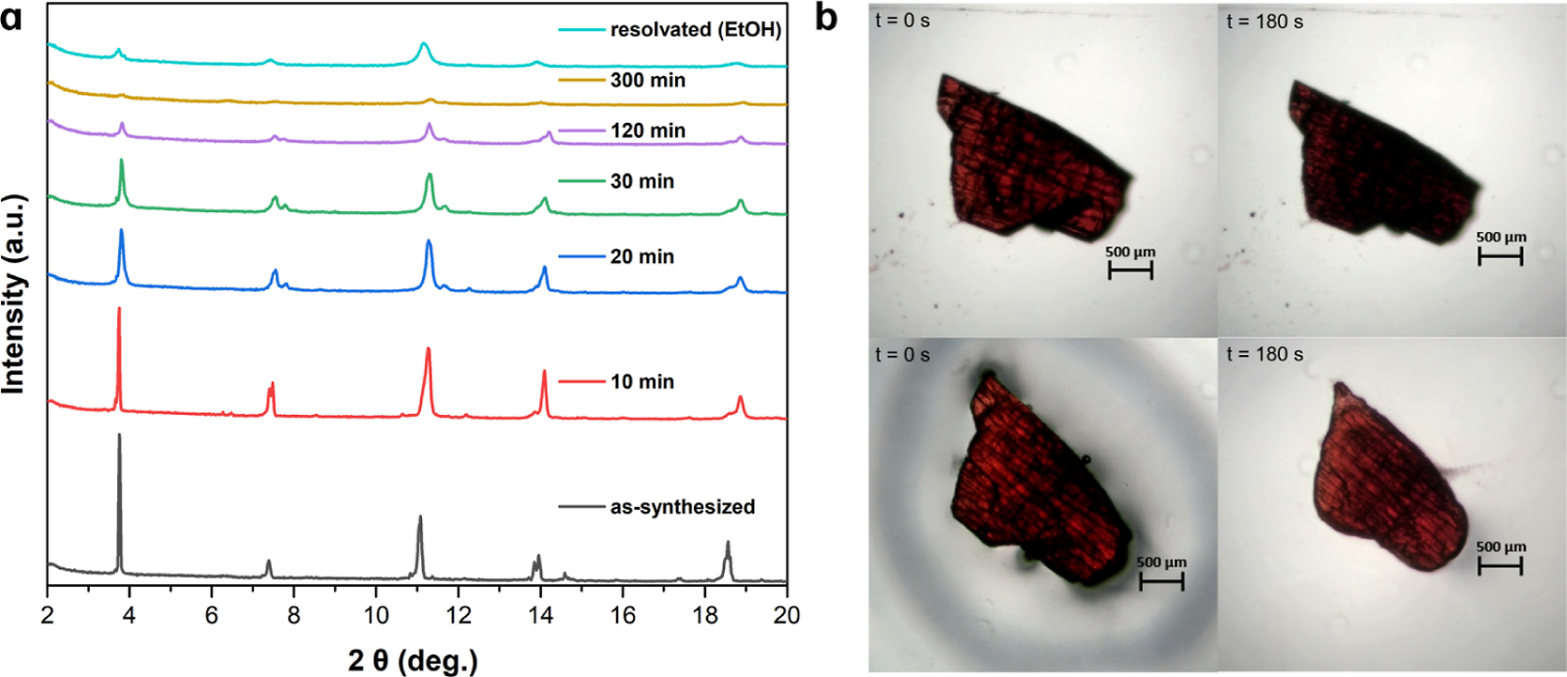
(a) Evolution of the PXRD pattern of as-synthesized **PTMTC**^**R@NR**^**-Zn MORF** over time and PXRD of a resolvated material with ethanol. (b) Optical microscopic images of a **PTMTC**^**R@NR**^**-Zn MORF** crystal. In the top image: the crystal was initially in contact with ethanol (0 s), and upon complete evaporation (at 180 s), its area had reduced by 10–15%. In the bottom image: the same crystal was subjected to a drop of ethanol, causing it to swell once again (0 s). Following this, the crystal contracted again upon complete evaporation of the ethanol (at 180 s).

Powder X-ray diffraction (PXRD) patterns of the as-synthesized **PTMTC**^**R@NR**^**-Zn MORF** were measured over time to study possible structural changes after desolvation (Figure 6a). The crystallinity gradually decreases as the solvent evaporates until the material becomes essentially amorphous after 300 min. Interestingly, crystallinity was partially recovered after resolvation with ethanol, showing an analogous behavior to what was previously observed in a similar PTMTC-based 2D MORF.^17^ The solvent-induced swelling behavior of **PTMTC**^**R@NR**^**-Zn MORF** was further studied by following the time evolution of a few single crystals removed from the solution using an optical microscope (Figures 6b and S29–S31). After removal from the solution, single crystals of **PTMTC**^**R@NR**^**-Zn MORF** crystals shrink rapidly upon solvent loss with an area reduction of ∼15% after 180 s (Figures 6b and S29). To verify the reversibility of the solvent-induced breathing phenomenon, a droplet of ethanol was applied to the same crystal. Initially, the crystal expanded until it quickly contracted once the solvent had evaporated (within 180 s) (Figure 6b, bottom). The procedure was repeated, with identical behavior observed, and area reduction/expansion rates ranged between 5 and 15% (see Figures S30 and S31). This confirms the reversibility of the process. This “breathing” behavior together with the gradual loss and recovery of crystallinity is analogous to that observed in the Cu_3_(PTMTC)_2_(py)_6_(CH_3_CH_2_OH)_2_(H_2_O) material reported by Maspoch, Veciana, and co-workers.^17^ This suggests that the reversible breathing/shrinking process after ethanol adsorption/evaporation is responsible for the structural changes. Thermogravimetric analysis (TGA) shows that **PTMTC**^**R@NR**^**-Zn MORF** is thermally stable up to ∼200 °C (Figure S32), whereas the N_2_ adsorption isotherm at 77 K revealed a minimum uptake of N_2_ which is likely related to the increased interlayer disorder after solvent evacuation. PTM-based MOFs exhibiting higher thermal and chemical stabilities could be designed by combining organic radical linkers with high-valent metals (Fe^3+^, Zr^4+^, Ti^4+^, etc.).

### 2.7 Luminescence Properties of **PTMTC**^**R@NR**^**-Zn MORF**

**Figure 7:**
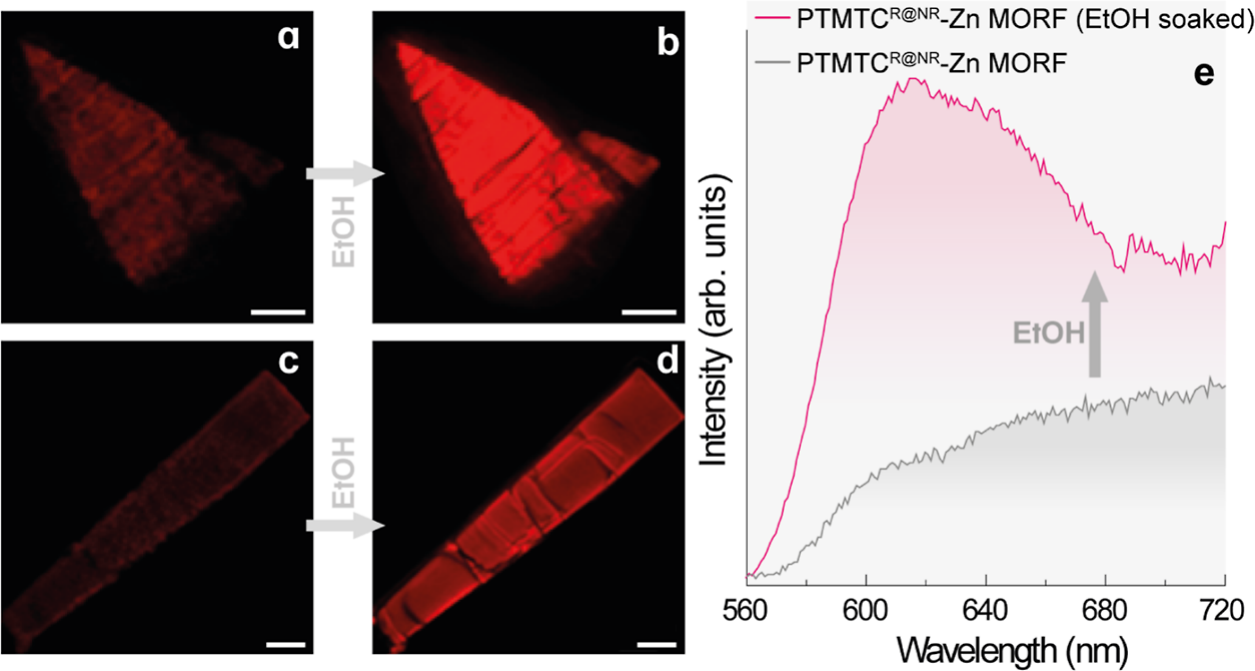
Confocal microscopic images of (a,c) dried and (b,d) ethanol-soaked crystals of **PTMTC**^**R@NR**^**-Zn** MORF illuminated with 400 nm light at room temperature. Scale bars correspond to 20 μm. (e) Emission spectra upon 377 nm excitation of a single crystal of **PTMTC**^**R@NR**^**-Zn** MORF soaked in ethanol and after evaporation at room temperature.

The emission spectra (λ_exc_ = 377 nm) of the **PTMTC**^**NR**^**-Zn MOF** and **PTMTC**^**R@NR**^**-Zn MORF** solids were recorded at room temperature (Figure S33). The **PTMTC**^**R@NR**^**-Zn MORF** emission spectrum shows an intense band at 610 nm in agreement with the characteristic **PTMTC**^**R**^ emission band, while **PTMTC**^**NR**^**-Zn MOF** is nonemissive. To study the impact of the breathing behavior on the optical properties, images of both dried and ethanol-soaked crystals were taken using a confocal microscope while being illuminated with 400 nm light at room temperature (Figure 7a–d). Crystals of **PTMTC**^**R@NR**^**-Zn MORF** show enhanced luminescence when exposed to ethanol and then decreased upon ethanol evaporation. This variation in the emission intensity was confirmed by measuring the emission spectrum of a single crystal at room temperature (Figure 7e). The crystal freshly removed from the solvent shows an emission band peaking at 610 nm, which is in agreement with the emission band of **PTMTC**^**R**^ in solution and the solid state (Figure 1). The differences in the spectroscopic features are common when comparing the emission of thin films and single crystals.^40^ The emission of **PTMTC**^**R@NR**^**-Zn MORF** single crystals rapidly decreases in intensity as the ethanol evaporates (Figure S34). When soaking the material using alternative solvents (hexane, THF, and CH_2_Cl_2_), we observed a significantly reduced emission intensity, almost negligible in comparison. Only when using a similar solvent such as propanol, an analogous behavior was observed (Figure S35), suggesting a distinct selectivity for ethanol and propanol. The enhanced luminescence upon ethanol adsorption may be attributed to the crystalline solvated material positioning the **PTMTC**^**R**^ radical moieties in fixed positions, preventing quenching from exciton migration among proximate radicals.^10^ Photostability studies on the **PTMTC**^**R**^ ligand suggest that the luminescence decrease in the desolvated material is not a result of radical decomposition. PLQY of **PTMTC**^**R@NR**^**-Zn MORF** was found to be 2.4 ± 0.2% (upon 377 nm excitation) when the radical content is around ∼15% and slightly higher (3.2 ± 0.2%) when using small amounts of radical (1–2%). This value is consistent with that of the **PTMTC**^**R**^**@PTMTC**^**NR**^ thin film with 1% radical concentration (see above). This study demonstrates that the luminescent properties of **PTMTC**^**R@NR**^**-Zn MORF** can be modulated by varying the concentration of the **PTMTC**^**R**^ radical linker in the framework or by applying an external stimulus, such as exposure to solvents. In addition, the effect of the magnetic field on luminescence could also be studied to explore a possible magnetoluminescence behavior, as observed in some similar luminescent organic radicals.^21,41^

## 3 Conclusions

This work has presented a detailed exposition on the synthesis of a novel multivariate metal–organic radical framework, referred to as **PTMTC**^**R@NR**^**-Zn MORF**, through the combination of both open-shell and closed-shell organic ligands with Zn^2+^ metal centers. A key finding is the preservation of luminescence from organic radicals. This is facilitated by the effective isolation of radical centers within the framework. In addition, the magnetic and electrochemical properties of the organic radical were also preserved within the multivariate framework, whereas the presence of both closed- and open-shell organic linkers was further confirmed by KPFM measurements.

A salient feature of this newly synthesized material is its layered crystal structure, which features 1D microporous channels within its layers. A distinct characteristic of **PTMTC**^**R@NR**^**-Zn MORF** is its reversible swelling, capable of reaching 15% in response to ethanol adsorption. An intriguing concomitant phenomenon observed during ethanol adsorption is the activation of luminescence.

The insights gleaned from this study possess potential implications for the future design of stimuli-responsive materials. Furthermore, these results could engender the creation of hybrid materials sparingly doped with trace amounts of organic radicals. Such a design could provide a controlled modulation of their optical, magnetic, and electrochemical properties, which would be of great interest when combining such properties, synergistically (e.g., magnetoluminescence) or for potential applications in sensing, energy storage, or electrocatalysis. In essence, the implications of this study extend to the overarching realm of materials science and suggest exciting avenues for future research.

## 4 Experimental Section

### 4.1 General Methods and Materials

All reagents and solvents employed in the syntheses were of high-purity grade and were purchased from Sigma-Aldrich Co. or TCI. ^1^H liquid-state NMR spectra were recorded on a Bruker AVANCE 300 spectrometer (300 MHz). Tetramethylsilane was used as an internal reference. Chemical shifts (δ) are quoted in parts per million from TMS and the coupling constants (*J*) in Hz. Positive-ion ESI mass spectra were acquired using a Q-TOF 2 instrument. Nitrogen was used as a nebulizer gas and argon as a collision gas. The needle voltage was set at 3000 V, with the ion source at 80 °C and desolvation temperature at 150 °C. The cone voltage was 35 V. Infrared spectra were recorded using powdered samples in an ATR FT-IR GALAXY SERIES FT-IR 7000 (Mattson Instruments) spectrometer in the 4000–400 cm^–1^ range. Powder X-ray diffraction patterns were recorded using an Empyrean PANalytical diffractometer, equipped with an PIXcel 1D detector and a flat-plate sample holder in a Bragg-Brentano para-focusing optics configuration (45 kV, 40 mA). EPR measurements were performed in an EMX 300 equipment (Bruker) at room temperature. Magnetic susceptibility measurements were performed using an MPMS3 SQUID-VSM Magnetometer (7 T) (Quantum Design) or a PPMS-9 equipment (9 T) (Quantum Design). TGA was measured in a Q5000 IR thermobalance (TA Instruments). The UV-vis absorption spectra were measured with a Jasco UV-660 Spectrophotometer (Jasco International, Tokyo). KPFM measurements were done at rarefied atmosphere using Park NX HiVac microscope (Park Systems) and a SSS-NCHR probe (Nanosensors) with the tip curvature radius below 2 nm.

### 4.2 Photoluminescence Measurements

The emission and excitation spectra were recorded on a modular double-grating excitation spectrofluorometer with a TRIAX 320 emission monochromator (Fluorolog-3, Horiba Scientific) coupled to a near-infrared R928 Hamamatsu photomultiplier using the front face acquisition mode. The excitation source was a 450 W Xe arc lamp. Both recorded emission and excitation spectra were corrected with the spectrofluorometer optical spectral response and the spectral distribution of the lamp intensity using a photodiode reference detector, respectively. Absolute PLQYs were measured with a quantum yield measurement system Quantaurus-QY (C13534, Hamamatsu), equipped with a 150 W xenon lamp coupled to a monochromator for wavelength discrimination, an integrating sphere as the sample chamber, and two multichannel analyzers for signal detection in the visible and NIR spectral ranges. The excitation wavelength was 378 nm.

### 4.3 Electrochemical Measurements

The electrochemical experiments were performed using an Autolab electrochemical workstation (PGSTAT302N with FRA32 M Module) connected to a personal computer that uses Nova 2.1 electrochemical software. A typical three-electrode experimental cell equipped with a platinum wire as the counter electrode and a silver wire as the pseudoreference electrode was used for the electrochemical characterization of the working electrodes. The electrochemical properties were studied by measuring the cyclic voltammogram at different scan rates in a previously N_2_ purged 0.1 M TBAPF_6_/CH_2_Cl_2_ solution. Ferrocene was added as an internal standard upon completion of each experiment. All potentials are reported in V versus Ag/AgCl. Electrode preparation: The powdered materials (2 mg) were mixed in 2 mL of Nafion and ethanol (1:3). 100 μL was deposited on a 3 mm diameter glassy carbon disc working electrode, which was previously polished with 0.3, 0.1, and 0.05 μm alumina powders. Afterward, the solvent was evaporated at room temperature.

## Supporting Information Available

General methods and materials, synthesis and characterization of **PTMTC**^**R**^, preparation of **PTMTC**^**R**^**@PTMTC**^**NR**^ films, photostability studies, synthesis and crystal structures of **PTMTC**^**NR**^**-Zn MOF** and **PTMTC**^**R@NR**^**-Zn MORF**, KPFM measurements, breathing behavior, and optical properties of **PTMTC**^**R@NR**^**-Zn MORF**(PDF)
Crystal structure of **PTMTC**^**NR**^**-Zn MOF** (CIF)
Crystal structure of **PTMTC**^**R@NR**^**-Zn MORF** (CIF)
Breathing behavior of **PTMTC**^**R@NR**^**-Zn MORF** (AVI)

## Supplementary Material

cm3c02460_si_001.pdf

cm3c02460_si_002.cif

cm3c02460_si_003.cif

cm3c02460_si_004.avi

## Abbreviations

CV: cyclic voltammetry
MOF: metal–organic framework
PLQY: photoluminescence quantum yield
PTM: perchlorotriphenylmethyl
PTMTC: perchlorotriphenylmethyl tricarboxylic acid
PXRD: powder X-ray diffraction
