# Supplementary material for: Exploring the Luminescence, Redox, and Magnetic Properties in a Multivariate Metal–Organic Radical Framework
Source: Chem Mater. 2024 Jan 23;36(3):1333–41. doi: 10.1021/acs.chemmater.3c02460 (PMC10870702; doi:10.1021/acs.chemmater.3c02460)
Supplement: Supplementary file 1 — cm3c02460_si_001.pdf [file cm3c02460_si_001.pdf]

Supporting Information

Exploring the Luminescence, Redox and Magnetic Properties in a Multivariate Metal-Organic Radical Framework

Gonçalo Valente,[†] Pedro Ferreira,[†] Miguel A. Hernández-Rodríguez,[‡] Carlos D. S. Brites,[‡] João Amaral,[‡] Pavel Zelenovskii,[‡] Filipe A. Almeida Paz,[†] Samuel Guieu,[†,*] João Rocha,[†,*] Manuel Souto[†,§,*]

[†] Department of Chemistry, CICECO-Aveiro Institute of Materials, University of Aveiro, Aveiro, 3810-393, Portugal

[‡] Department of Physics, CICECO-Aveiro Institute of Materials, University of Aveiro, Aveiro, 3810-393, Portugal

[§] CIQUS, Centro Singular de Investigación en Química Bioloxica e Materiais Moleculares, Departamento de Química-Física, Universidade de Santiago de Compostela, 15782, Santiago de Compostela, Spain

Contents

1. **General methods and materials**
2. **Synthesis and characterization of PTMTC^{R}**
3. **Preparation of PTMTC^{R}@PTMTC^{NR} films.**
4. **Photostability studies**
5. **Synthesis of PTMTC^{NR}-Zn MOF and PTMTC$^{R@NR}$-Zn MORF**
6. **Crystal structure of PTMTC^{NR}-Zn MOF and PTMTC$^{R@NR}$-Zn MORF**
7. **Kelvin probe force microscopy (KPFM)**
8. **Breathing behavior and optical properties of PTMTC$^{R@NR}$-Zn MORF**
9. **References**

1. General methods and materials

All reagents and solvents employed in the syntheses were of high purity grade and were purchased from Sigma-Aldrich Co., or TCI. ^{1}H liquid-state NMR spectra were recorded on a Bruker AVANCE 300 spectrometer (300 MHz). Tetramethylsilane was used as an internal reference. Chemical shifts (δ) are quoted in ppm from TMS and the coupling constants (*J*) in Hz. Positive-ion ESI mass spectra were acquired using a Q-TOF 2 instrument [Nitrogen was used as nebulizer gas and argon as collision gas. The needle voltage was set at 3000 V, with the ion source at 80 °C and desolvation temperature at 150°C. The cone voltage was 35 V]. Infrared spectra were recorded using powdered samples in an ATR FT-IR GALAXY SERIES FT-IR 7000 (Mattson Instruments) spectrometer in the 4000-400 cm^{-1} range. EPR measurements were performed in a EMX 300 equipment (Bruker) at room temperature. Magnetic susceptibility measurements were performed using a MPMS3 SQUID-VSM Magnetometer (7 Teslas) (Quantum Design) or PPMS-9 equipment (9 Teslas) (Quantum Design). TGA was measured in a Q5000 IR thermobalance (TA instruments).

Photoluminescence spectroscopy: The emission and excitation spectra were recorded on a modular double grating excitation spectrofluorimeter with a TRIAX 320 emission monochromator (Fluorolog-3, Horiba Scientific) coupled to a near-infrared R928 Hamamatsu photomultiplier, using the front face acquisition mode. The excitation source was a 450 W Xe arc lamp. Both recorded emission and excitation spectra were corrected with the spectrofluorimeter optical spectral response and the spectral distribution of the lamp intensity using a photodiode reference detector, respectively. Absolute photoluminescence quantum yields (PLQY) were measured with a quantum yield measurement system Quantaurus-QY (C13534, Hamamatsu), equipped with a 150 W Xenon lamp coupled to a monochromator for wavelength discrimination, an integrating sphere as sample chamber and two multi-channel analyzers for signal detection in the visible and in the NIR spectral ranges. The excitation wavelength was 378 nm.

Electrochemical measurements: The electrochemical experiments were performed using an Autolab electrochemical workstation (PGSTAT302N with FRA32M Module) connected to a personal computer that uses Nova 2.1 electrochemical software. A typical three-electrode experimental cell equipped with a platinum wire as the counter electrode and a silver wire as the pseudoreference electrode was used for the electrochemical characterization of the working electrodes. The electrochemical properties were studied measuring the cyclic voltammogram at different scan rates in previously N_2 purged 0.1 M $TBAPF_6/CH_2Cl_2$ solution. Ferrocene was added as an internal standard upon completion of each experiment. All potentials are reported in V versus Ag/AgCl. Electrode preparation: The powdered materials (2 mg) were mixed in 2 mL of Nafion and ethanol (1:3). 100 µL were deposited on a 3 mm diameter glassy carbon disc working electrode, which was previously polished with 0.3, 0.1, and 0.05 µm alumina powders. Afterwards, the solvent was evaporated at room temperature.

2. Synthesis of $PTMTC^R$

First, tris(2,3,5,6-tetrachlorophenyl)methane (**1**) was synthesized via Friedel-Crafts alkylation of 1,2,4,5-tetrachlorobenzene with $CHCl_3$ in the presence of $AlCl_3$. Then the reaction of **1** with *n*-BuLi and tetramethylethylenediamine (TMEDA) in anhydrous THF at low temperature formed the corresponding trianion that subsequently reacted with ethyl chloroformate to give the perchlorotriphenylmethyl triester **2**. Hydrolysis of ester **2** with concentrated sulfuric acid at 90 ºC yielded the non-radical perchlorotriphenyl tricarboxylic acid **$PTMTC^{NR}$** as a white powder (75 % yield). Finally, subsequent deprotonation of **$PTMTC^{NR}$** with NaOH followed by oxidation of the corresponding carbanion with I_2 afforded **$PTMTC^R$** as a red powder (80 % yield), whose formation was monitored by UV-vis spectroscopy (Figures S9-S10).

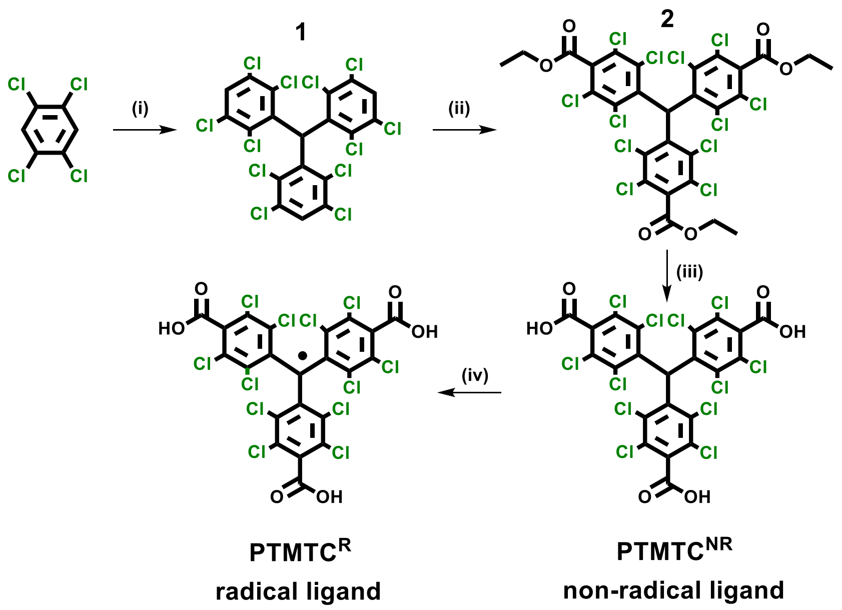

Scheme S1. Synthesis of **$PTMTC^R$**. (i) $AlCl_3$, $CHCl_3$, 165ºC, 24h, 67%; (ii) TMEDA, *n*-BuLi, THF, -78ºC, 1h; then ethyl chloroformate, 61%; (iii) conc. H_2SO_4, 90ºC, 12h, 75%; (iv) NaOH, DMSO, RT, 72h, then I_2, 80%.

2.1. Synthesis of tris(2,3,5,6-tetrachlorophenyl)methane (1)

1

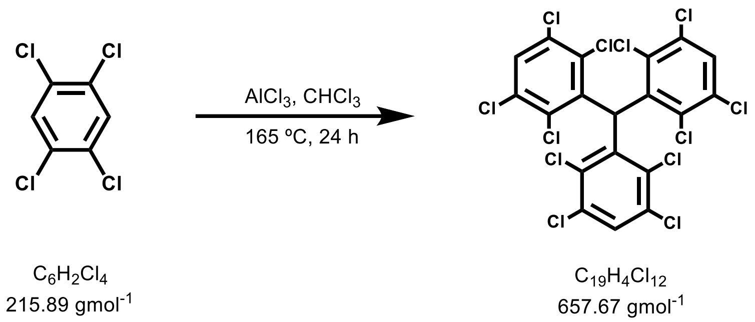

Scheme S2. Synthesis of tris(2,3,5,6-tetrachlorophenyl)methane (**1**).

1,2,4,5-tetrachlorobenzene (9.6 g, 44 mmol) was mixed with aluminum chloride ($AlCl_3$) (1.66 g, 12.48 mmol) and chloroform ($CHCl_3$) (1.0 mL, 12.48 mmol) in a glass pressure vessel. The mixture was heated at 165 °C for 24 hours. Then, the mixture was cooled with ice and hydrochloric acid (HCl) (1 M, 50 mL) was added. The mixture was extracted three times with $CHCl_3$. The organic layer was then washed with water and aqueous sodium bicarbonate ($NaHCO_3$) dried over sodium sulfate (Na_2SO_4) and the solvent was removed by evaporation under reduced pressure. The residue was purified by flash chromatography on silica gel using hexane as an eluent. The result of the purification process yielded 2.15 g of white powder (67 % yield) (**1**). Characterization: ^{1}H-NMR (400 MHz, $CDCl_3$), δ (ppm): 7.65 (s, 3H), 6.98 (s, 1H). IR (cm^{-1}): 3116, 3068, 2929, 1728, 1547, 1410, 1386, 1346, 1322, 1238, 1198, 1161, 1097, 973, 864, 850, 780, 704, 688, 647, 623, 559, 539, 483.

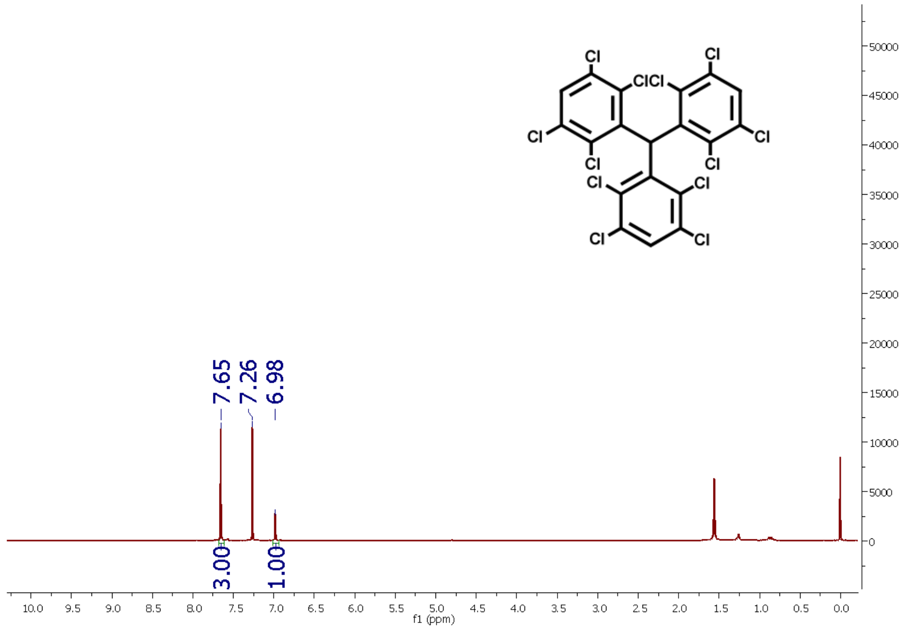

Figure S1. ^{1}H-NMR spectrum of tris(2,3,5,6-tetrachlorophenyl)methane (**1**) in $CDCl_3$.

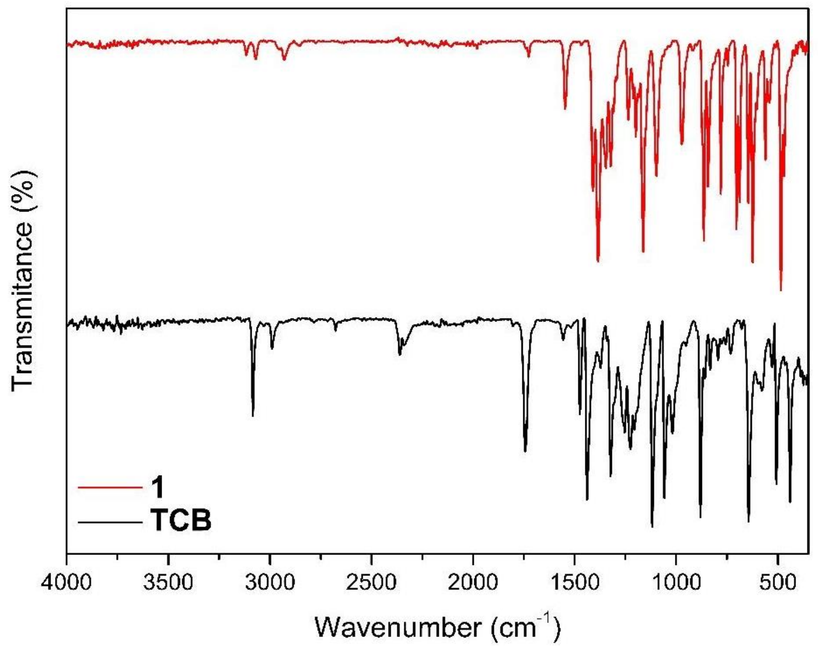

Figure S2. IR spectra of 1,2,4,5-Tetrachlorobenzene (TCB) and tris(2,3,5,6-tetrachlorophenyl)methane (**1**).

2.2. Synthesis of tris(4-ethoxycarbonyl-2,3,5,6-tetrachlorophenyl)methane (2)

1 **2**

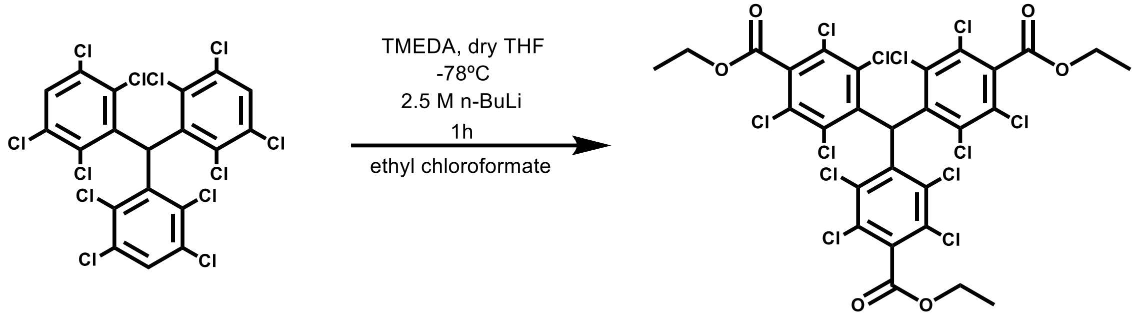

$C_{19}H_4Cl_{12}$
657.67 gmol^{-1}

$C_{28}H_{16}Cl_{12}O_6$
873.86 gmol^{-1}

Scheme S3. Synthesis of tris(4-ethoxycarbonyl-2,3,5,6-tetrachlorophenyl)methane (**2**).

Tris(2,3,5,6-tetrachlorophenyl)methane (**1**) (500 mg, 0.76 mmol) and N,N,N’,N-Tetramethylethylenediamine (TMEDA) (1.15 ml, 7.6 mmol) were dissolved in 50 mL of anhydrous tetrahydrofuran (THF) under inert atmosphere and cooled to -78 °C. Then, a solution of 2.5 M n-BuLi in *n*-hexane (3.95 mL, 10.0 mmol) was added in a single step, and the mixture was stirred at this temperature for one hour. Subsequently, ethyl chloroformate (0.94 mL, 10.0 mmol) was added, and the reaction mixture was allowed to reach room temperature. After 16h, the solvent was evaporated, and the resulting residue was dissolved in dichloromethane (CH_2Cl_2). The organic layer was washed with water and dried with anhydrous Na_2SO_4. The solvent was removed under vacuum, and the remaining residue was purified over silica gel chromatography using CH_2Cl_2/hexane (1/1) as eluent to obtain 540 mg (81 % yield) of **2** as a white solid. Characterization: ^{1}H-NMR (400 MHz, $CDCl_3$), δ (ppm): 7.01 (s, 1H), 4.49 (q, J = 7.1 Hz, 6H), 1.43 (t, J = 7.1 Hz, 12H). IR (cm^{-1}): 3026-2840 (w, C-H), 1750 (s, C=O), 1555, 1455, 1341, 1298, 1259, 1224, 1198, 1113, 850 (s, C-Cl), 756, 651.

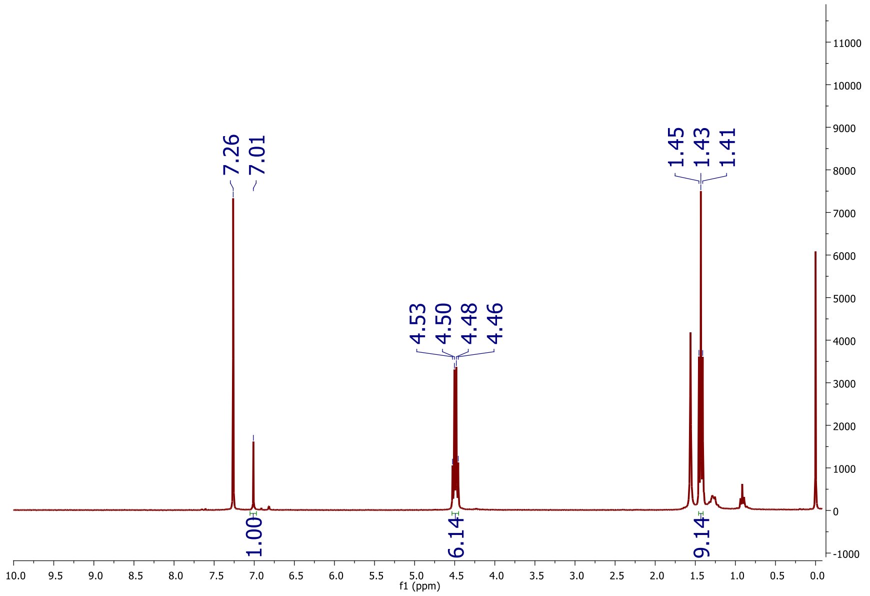

Figure S3. ^{1}H-NMR spectrum of tris(4-ethoxycarbonyl-2,3,5,6-tetrachlorophenyl)methane (**2**) in $CDCl_3$.

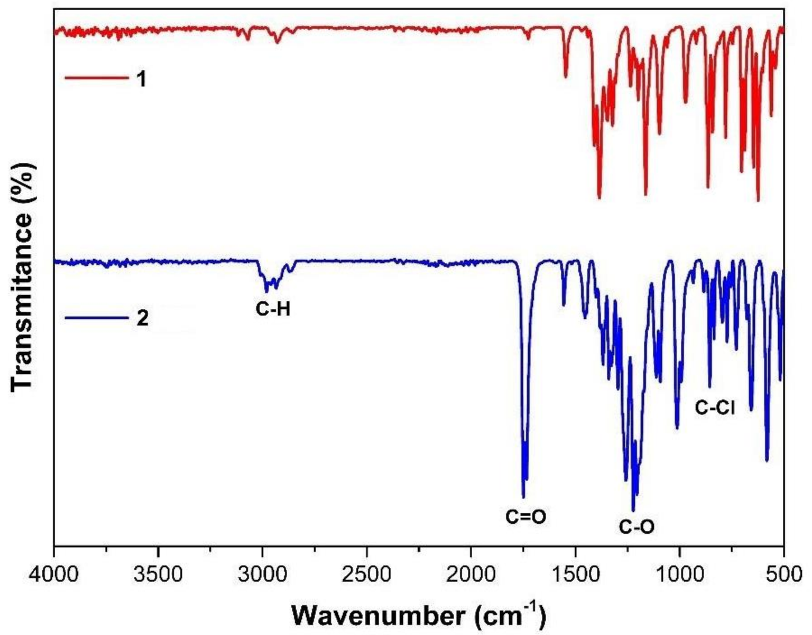

Figure S4. IR spectra of tris(2,3,5,6-tetrachlorophenyl)methane (**1**) and tris(4-ethoxycarbonyl-2,3,5,6-tetrachlorophenyl)methane (**2**).

2.3. Synthesis of tris(4-carboxyl-2,3,5,6-tetrachlorophenyl)methane (PTMTC^{NR})

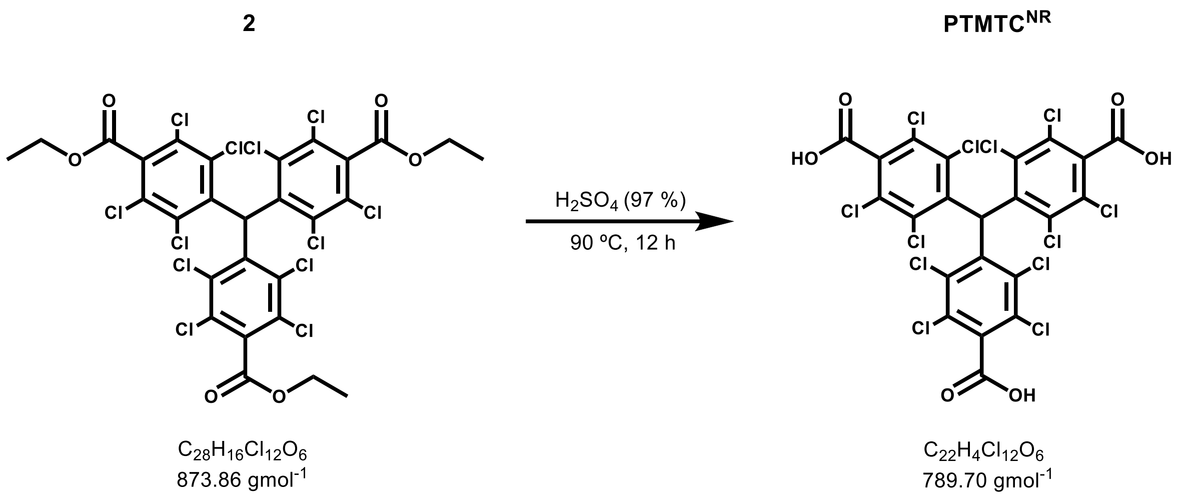

Scheme S4. Synthesis of tris(4-carboxyl-2,3,5,6-tetrachlorophenyl)methane (**PTMTC^{NR}**).

Compound **2** (250 mg, 0.29 mmol) was mixed with 30 mL of concentrated sulfuric acid (H_2SO_4) (97 %) and heated at 90 °C for 12 hours. Then, the mixture was cooled down to room temperature, ice was added, and the resulting aqueous phase was extracted with diethyl ether (Et_2O). The organic phase was concentrated and then extracted with an aqueous solution of Na_2CO_3. The resulting aqueous phase was acidified using 5 M HCl and extracted multiple times with Et_2O. The organic phase was dried with anhydrous Na_2SO_4, and the solvent was removed under vacuum. The crude product was dissolved in Et_2O and precipitated multiple times with hexane. **PTMTC^{NR}** was obtained as a white powder (200 mg, 88 % yield). Characterization: ^{1}H-NMR (400 MHz, DMSO-d$_6$), δ (ppm): 6.93 (s, 1H), 14.52 (s, 3H, Ar-COOH). ^{13}C-NMR (DMSO-d$_6$), δ (ppm): 164.28, 137.70, 137.51, 134.56, 133.84, 129.57, 128.61, 56.19. IR (cm^{-1}): 3020-2750 (w), 1672 (s, C=O), 1555, 1447, 1378, 1355, 1328, 1279, 1234, 1127, 885, 792, 715, 651, 526, 593.

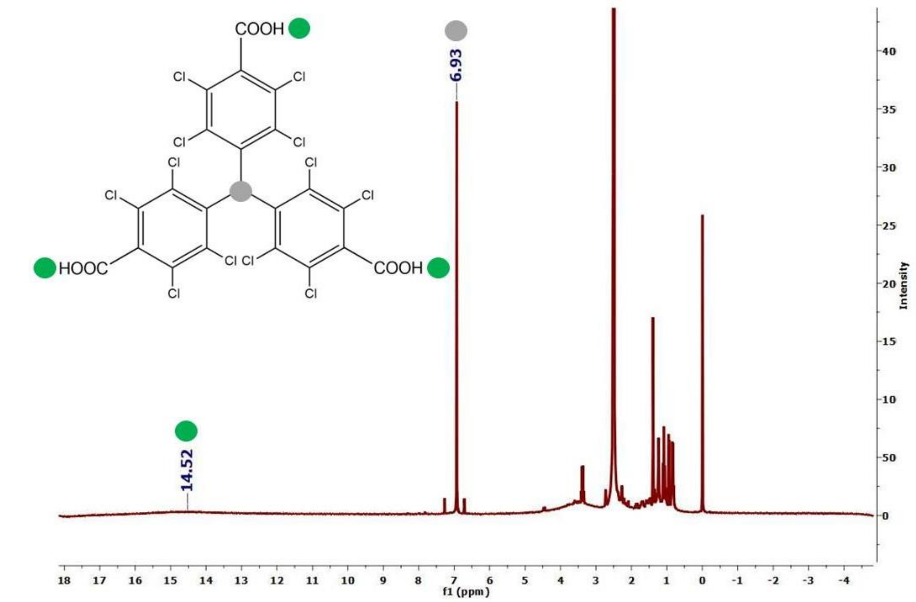

Figure S5. ^{1}H-NMR spectrum of tris(4-carboxyl-2,3,5,6-tetrachlorophenyl)methane (**PTMTC^{NR}**) in DMSO-d_6.

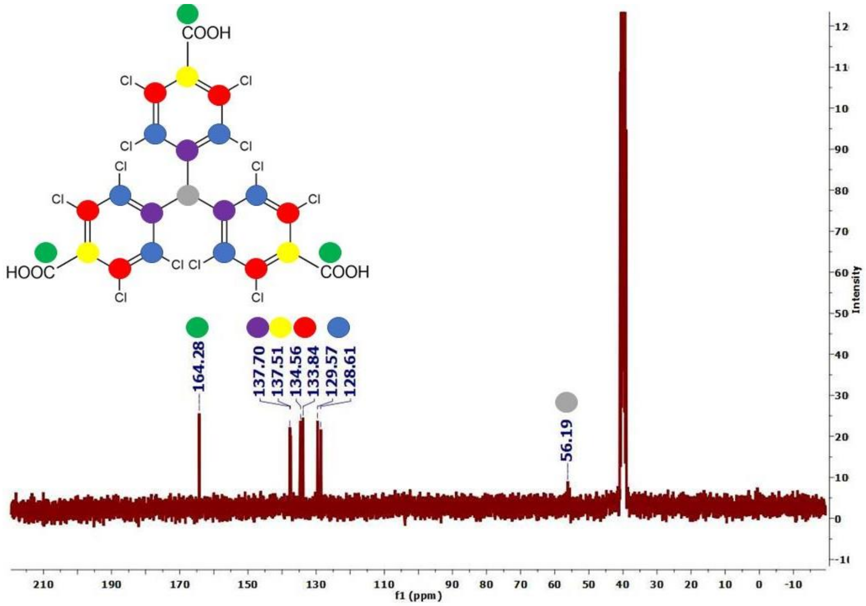

Figure S6. ^{13}C-NMR spectrum of tris(4-carboxyl-2,3,5,6-tetrachlorophenyl)methane (PTMTC^{NR}) in DMSO-d_6.

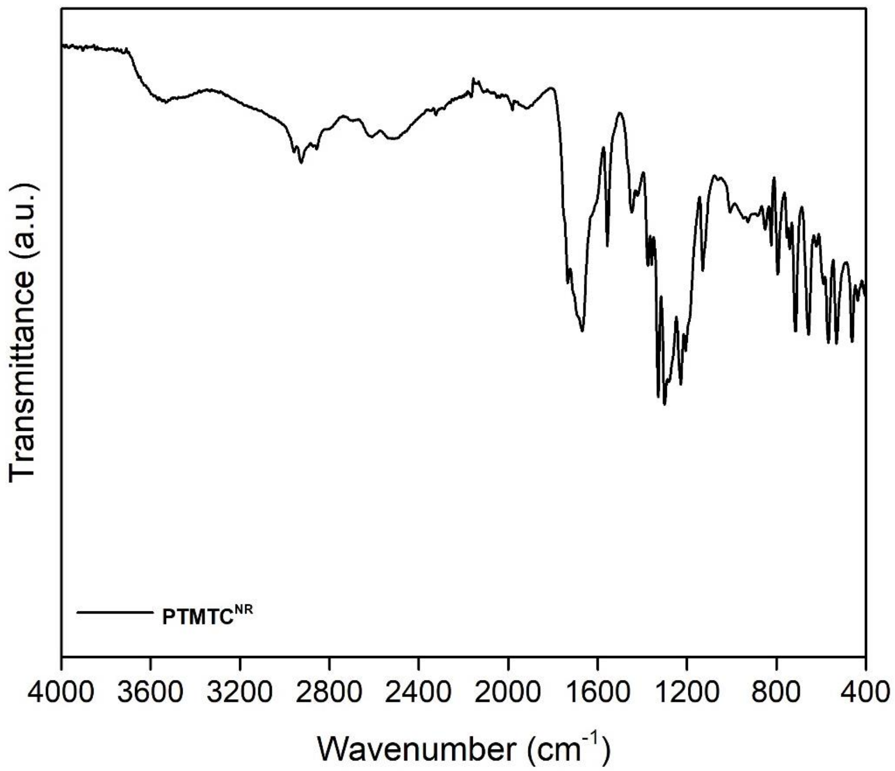

Figure S7. IR spectrum of **PTMTC^{NR}**.

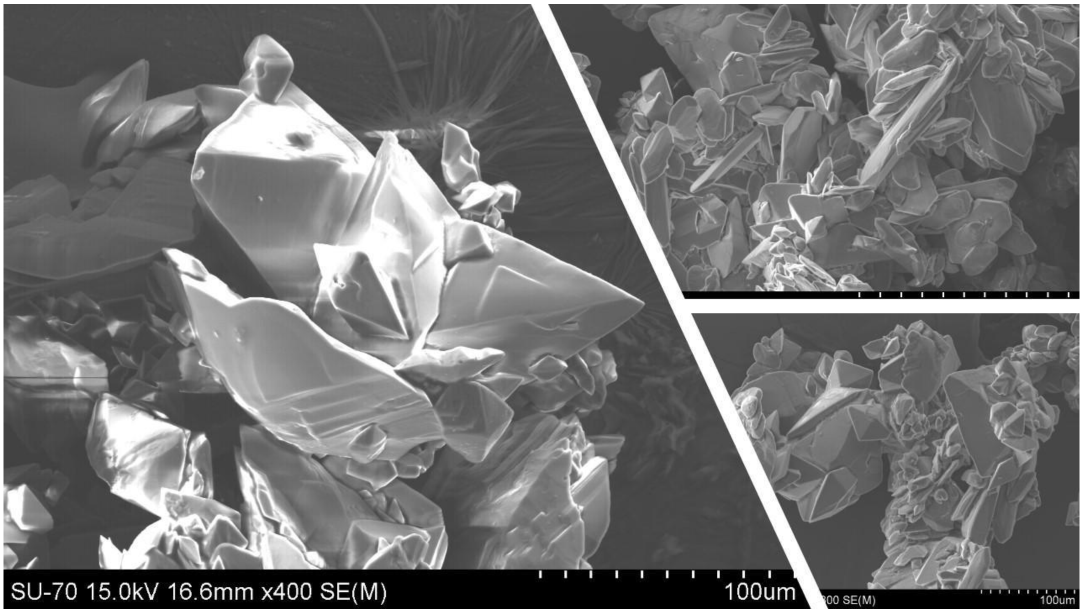

Figure S8. SEM images of tris(4-carboxyl-2,3,5,6-tetrachlorophenyl)methane (**PTMTC^{NR}**).

2.4. Synthesis of tris(4-carboxyl-2,3,5,6-tetrachlorophenyl)methyl radical (PTMTC^{R})

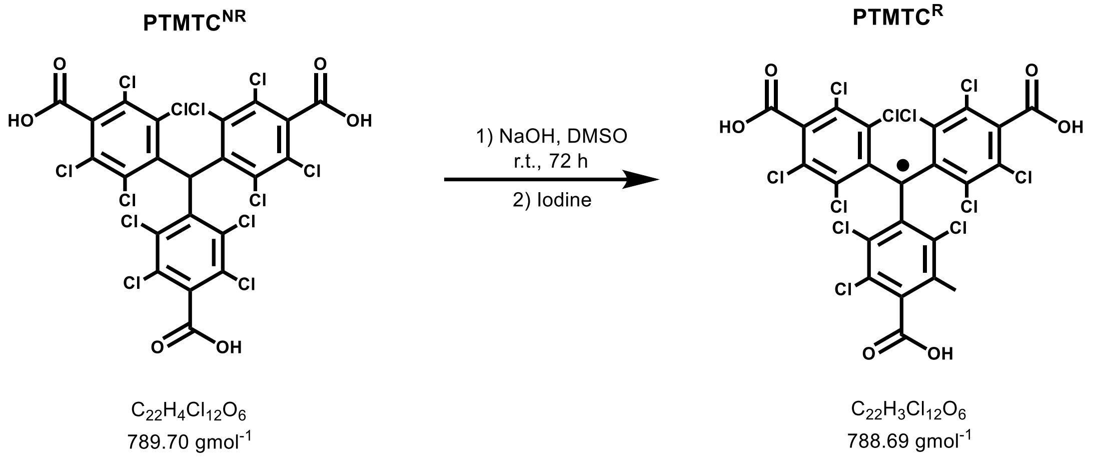

Scheme S5. Synthesis of radical tris(4-carboxyl-2,3,5,6-tetrachlorophenyl)methane radical (**PTMTC^{R}**).

Sodium hydroxide (700 mg, 17 mmol) was dissolved in 20 mL of DMSO. The resulting solution was sonicated and, after 1 hour, **PTMTC^{NR}** (200 mg, 0.25 mmol) was added to the mixture. The reaction was allowed to proceed for 72 hours in the dark with continuous stirring. The resulting mixture was filtered through cotton, and then iodine (127 mg, 0.50 mmol) was added to the reaction mixture. The reaction was left undisturbed in the dark and monitored by UV-vis spectroscopy until the formation of the radical was confirmed (absence of the characteristic band of anion at *ca.* 518 nm and appearance of the band at 378 nm). The product was extracted using an aqueous solution of sodium hydrogen carbonate ($NaHCO_3$) and subsequently acidified with 12 M HCl. Finally, the product was extracted with Et_2O and recrystallized from a mixture of Et_2O and *n*-pentane. This process yielded **PTMTC^{R}** (160 mg, 80 % yield) as a red powder. Characterization: IR (cm^{-1}): 2925, 2852, 1695 (C=O), 1540, 1437, 1351, 1218, 1041, 722. CV (*n*-Bu_4NPF_6 0.1 M in CH_2Cl_2 as electrolyte; V vs. Ag/AgCl): $E_{red}^{1/2}$ = -0.14 V. UV-Vis (in $CHCl_3$) λ (nm) (ε [M^{-1} cm^{-1}]): 378 (17000). ESI-TOF-MS (*m/z*): 787.3 [M^{-}]. EPR (CH_2Cl_2, 300 K): g = 2.003.

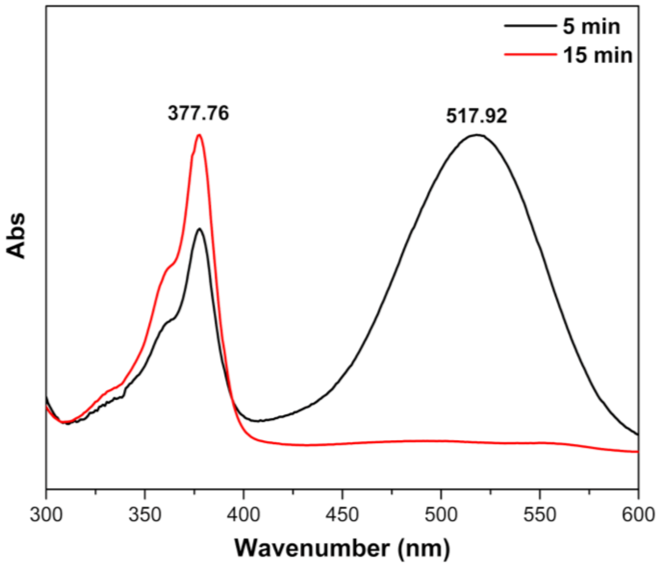

Figure S9. Evolution of the reaction for the synthesis of **PTMTC^R** monitored by UV-vis spectroscopy.

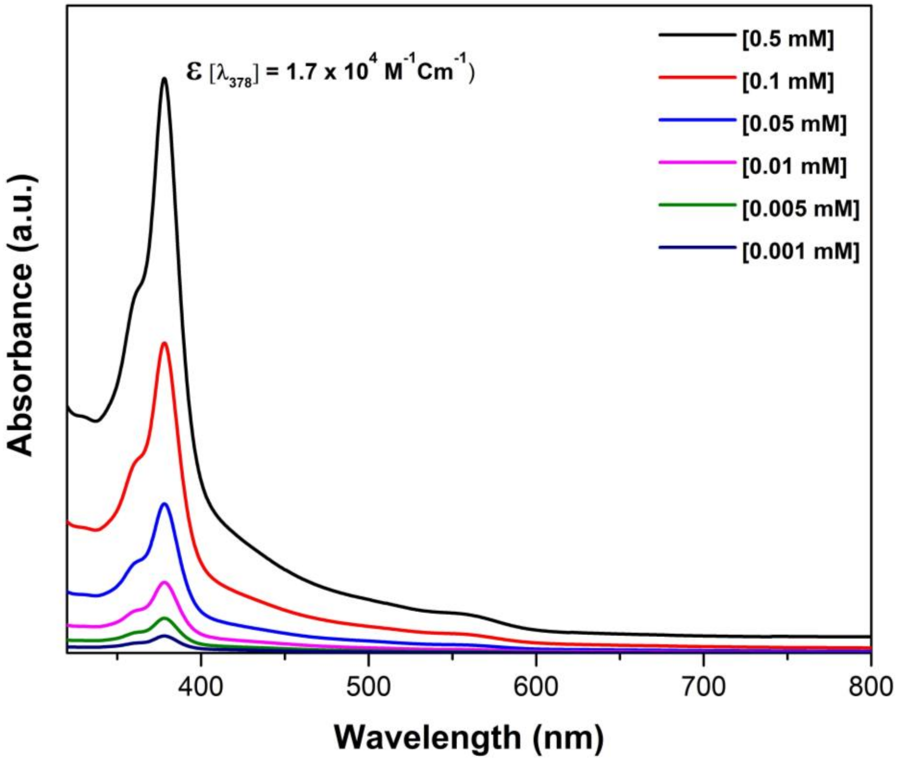

Figure S10. UV-vis spectra of the **PTMTC^R** at different concentrations in $CHCl_3$.

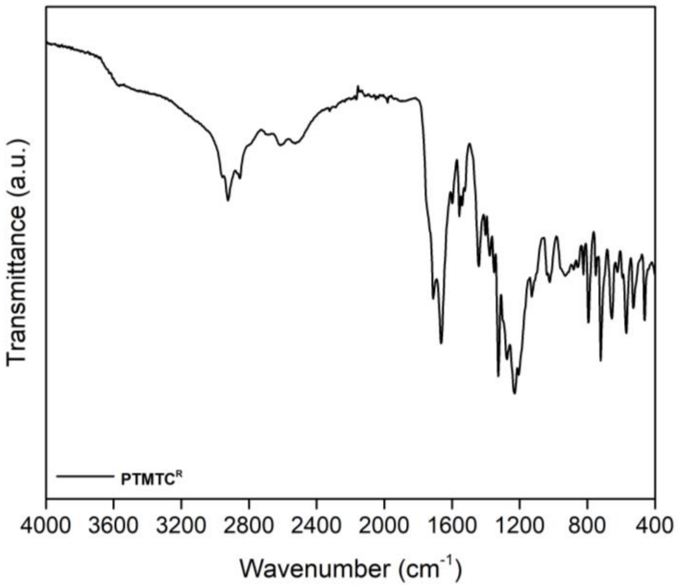

Figure S11. IR spectrum of **PTMTC^R**.

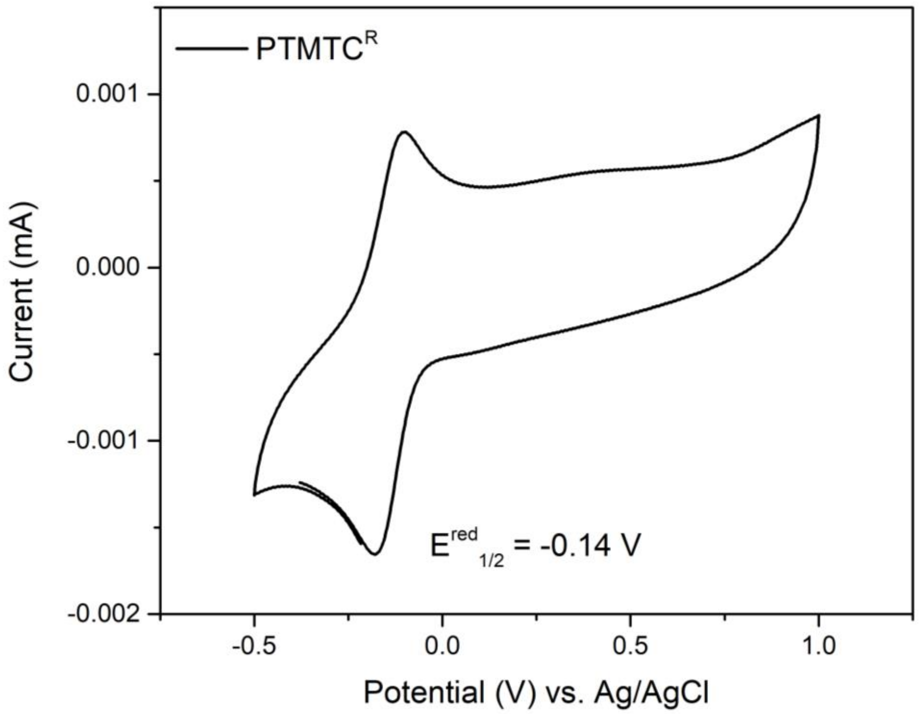

Figure 12. Cyclic voltammograms of **PTMTC^R** vs. Ag/AgCl using n-Bu_4NPF_6 in CH_2Cl_2 (0.1 M) as electrolyte at 0.1 V/s scan rate.

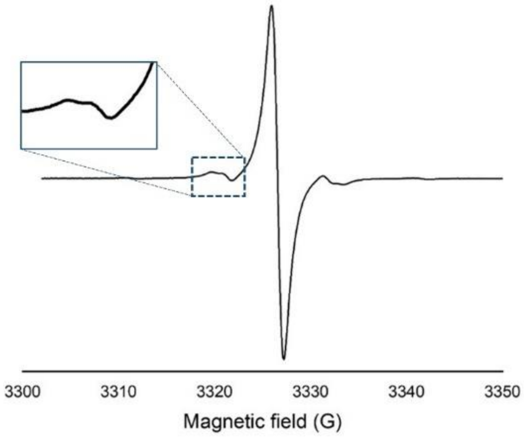

Figure S13. EPR spectra of **PTMTC^{R}** in CH_2Cl_2 (0.1 mM) at room temperature.

The temperature dependence of the magnetic susceptibility (χ) of **PTMTC^{R}** at ambient pressure was measured over the temperature range of 5−200 K. The compound shows a Curie−Weiss behavior and the experimental data was fitted to obtain a Curie constant C = 0.35 cm^3 K mol^{-1} with a χ_mT that is close to the theoretical value of 0.375 expected for noninteracting S = 1/2 systems.

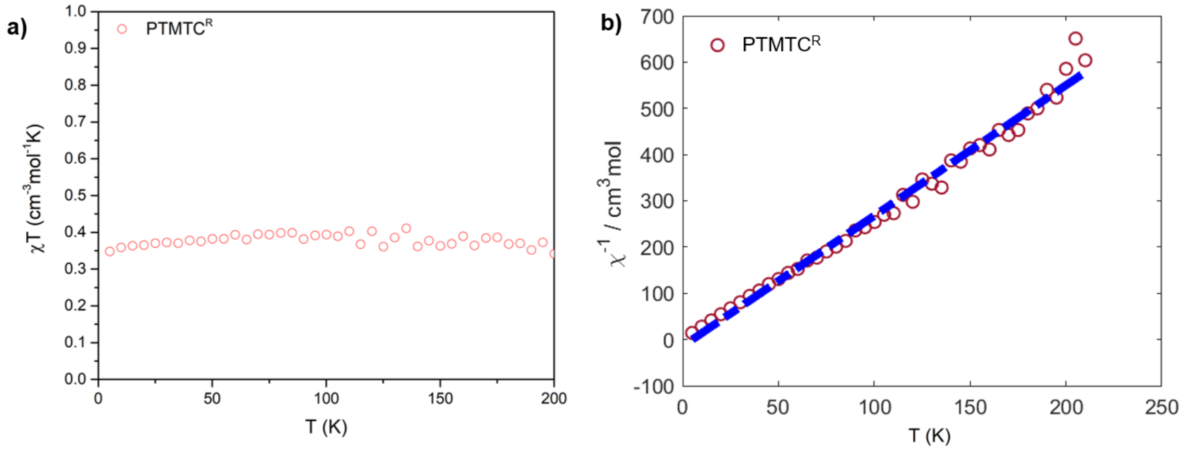

Figure S14. Magnetic susceptibility of **PTMTC^{R}** as a function of temperature in the 5-200 K range. a) $\chi \cdot$T versus T at H = 5000 Oe). b) χ^{-1} versus T and Curie-Wiess linear fit.

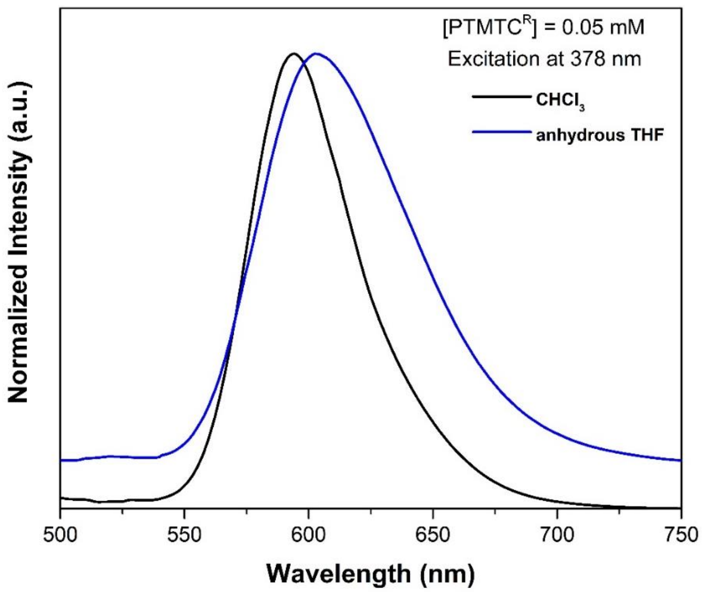

Figure S15. Emission spectra of the **PTMTC^{R}** (0.05 mM) in $CHCl_3$ and anhydrous THF excited at 378 nm.

3. Preparation of PTMTC^{R}@PTMTC^{NR} films.

Preparation of thin films with different radical concentration. **PTMTC^{R}@PTMTC^{NR}** films with different radical concentrations (1%, 2%, 3% and 4%) were prepared by spin coating in the dark. Solutions of 0.05 mM of **PTMTC^{R}@PTMTC^{NR}** in $CHCl_3$ were prepared and deposited on quartz substrates. The spin coating was performed at 1000 rpm for 1 minute. This procedure was repeated again to obtain 2 layers for each **PTMTC^{R}@PTMTC^{NR}** film.

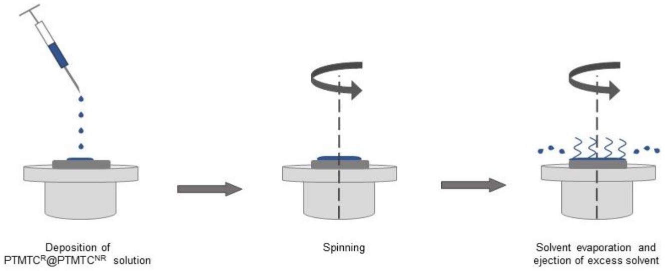

Radical concentration	Quantum Yield (± 10%)	Excitation wavelength
1%	0.032 ± 0.003 (3.2%)	378 nm
2%	0.027 ± 0.003 (2.7%)	378 nm
3%	0.022 ± 0.002 (2.2%)	378 nm
4%	0.018 ± 0.002 (1.8%)	378 nm

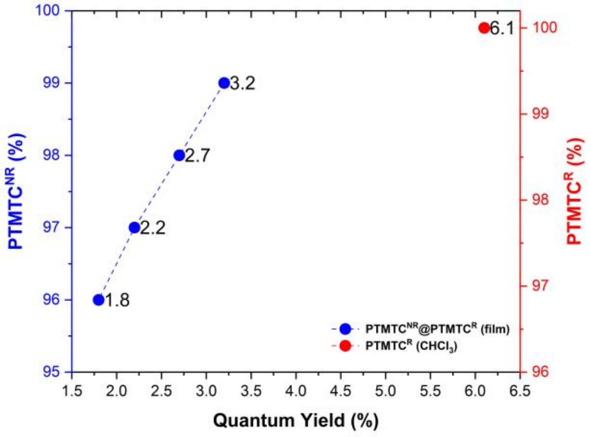

Figure S16. PLQY dependence on the concentration of **PTMTC^{R}** of **PTMTC^{R}@PTMTC^{NR}** films and PLQY of **PTMTC^{R}** in $CHCl_3$ (0.05 mM) as a reference.

4. Photostability studies

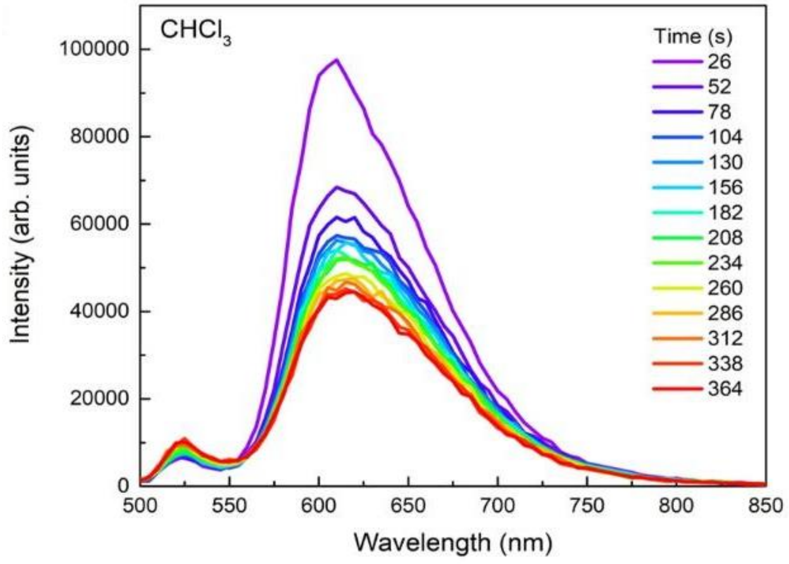

Figure S17. Photostability of **PTMTC^R** in $CHCl_3$. Evolution of the emission spectra over time exciting at λ = 378 nm.

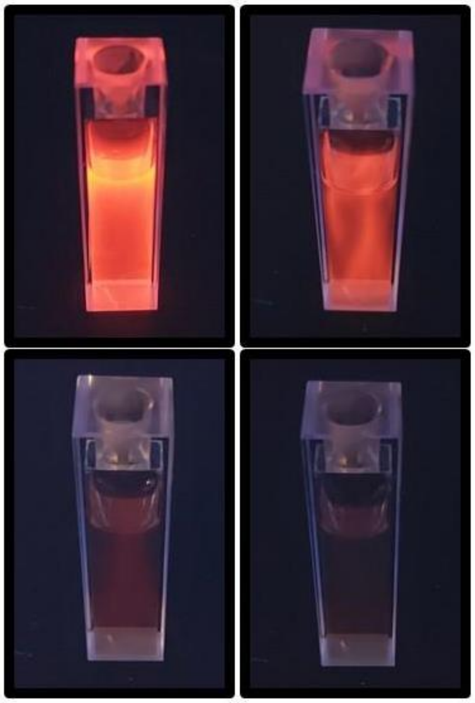

Figure S18. Pictures of **PTMTC^R** (0.05 mM) solution in $CHCl_3$ under constant UV irradiation taken every 10 min.

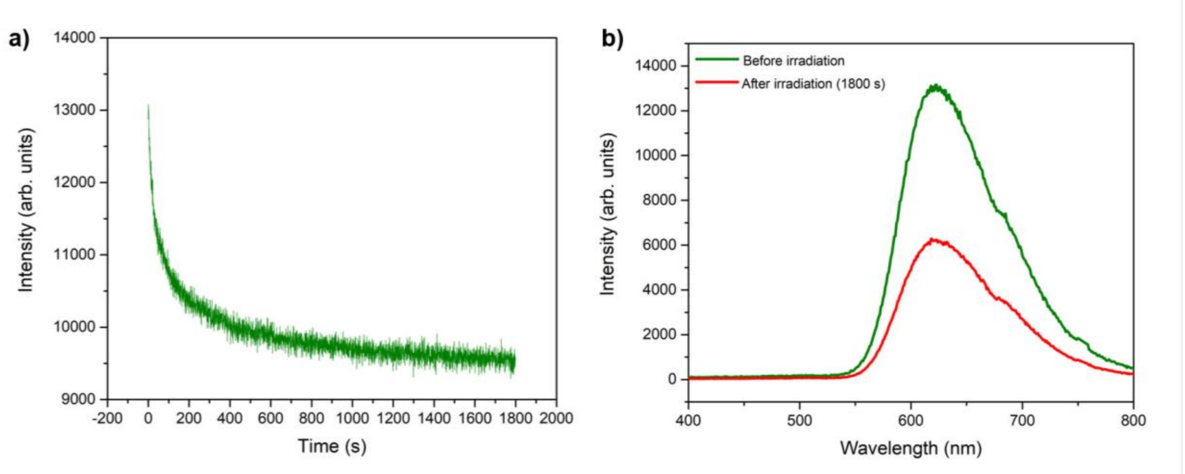

Figure S19. Photostability of **PTMTC^{R}@PTMTC^{NR}** (2/8) film deposited on a quartz substrate. a) Evolution of the emission intensity of **PTMTC^{R}@PTMTC^{NR}** (2/8) film deposited on quartz substrate over time. b) Emission spectra of **PTMTC^{R}@PTMTC^{NR}** (2/8) film deposited on a quartz substrate before and after irradiation (λ = 378 nm).

5. Synthesis and characterization of PTMTC^{NR}-Zn MOF and PTMTC$^{R@NR}$-Zn MORF

PTMTC^{NR} or **PTMTC^{NR}/PTMTC^{R}** (8/2) (8 mg, 0.0101 mmol) and zinc perchlorate hexahydrate [$Zn(ClO_4)_2 \bullet 6\ H_2O$] (5 mg, 0.0134) were weighed and mixed into a 4-mL flask. Then the mixture was dissolved in 2 mL of ethanol/water (3:1). After dissolution, 2 mL of ethanol/pyridine (7:1) was added carefully to the flask. After 15-20 days of slow evaporation, several white (**PTMTC^{NR}-Zn MOF**) or red (**PTMTC$^{R@NR}$-Zn MORF**) single crystals were formed (Figures S20-S21).

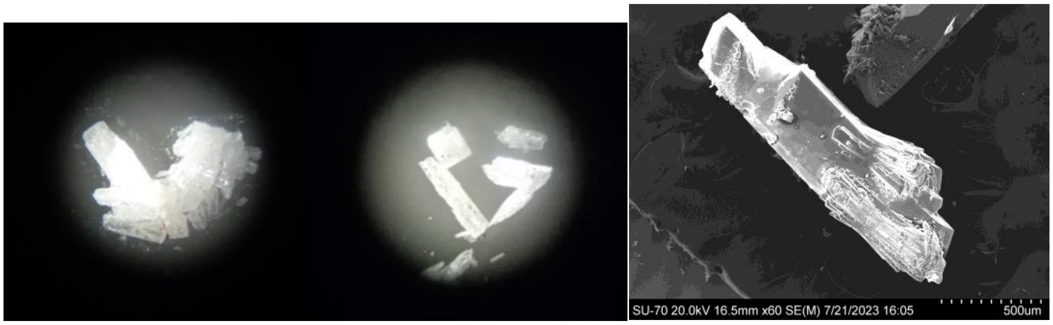

Figure S20. Optical microscope and SEM images of **PTMTC^{NR}-Zn MOF** crystals.

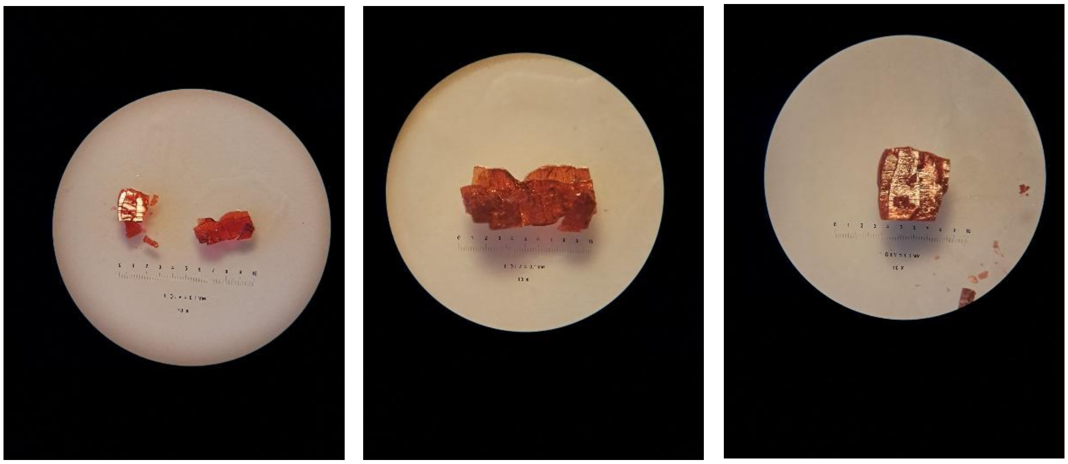

Figure S21. Images of **PTMTC$^{R@NR}$-Zn MORF** crystals taken using an optical microscope.

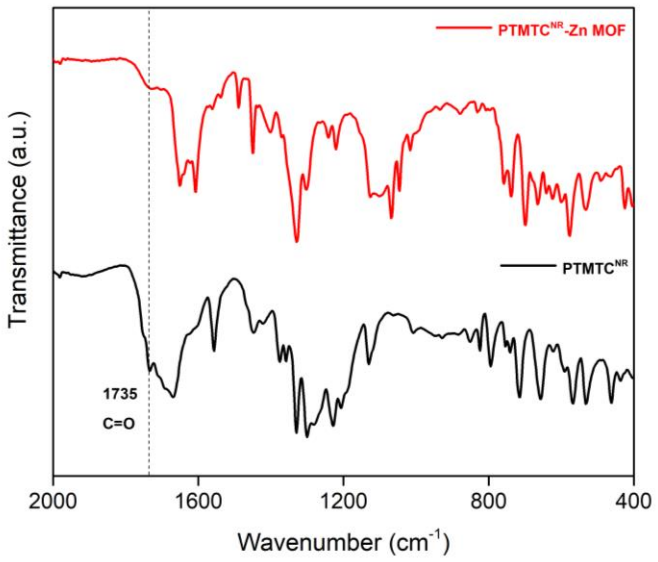

Figure S22. IR spectra of **PTMTC^{NR}** ligand and **PTMTC^{NR}-Zn MOF** crystals.

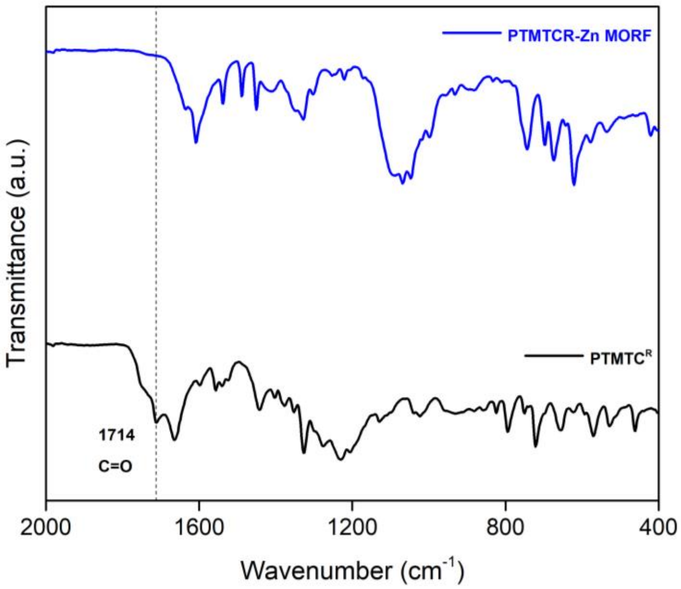

Figure S23. IR spectra of **PTMTC^{R}** ligand and **PTMTC$^{R@NR}$-Zn MORF** crystals.

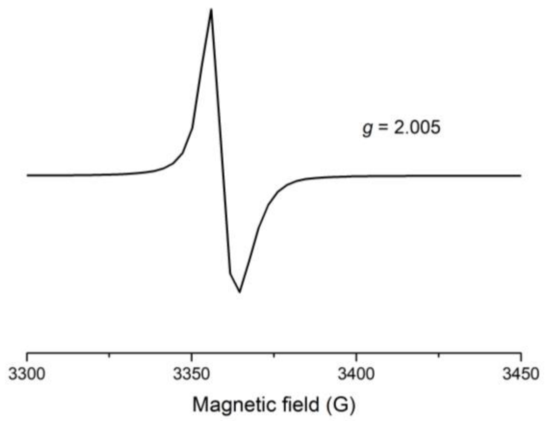

Figure S24. EPR spectrum of **PTMTC$^{R@NR}$-Zn MORF** single crystals at room temperature.

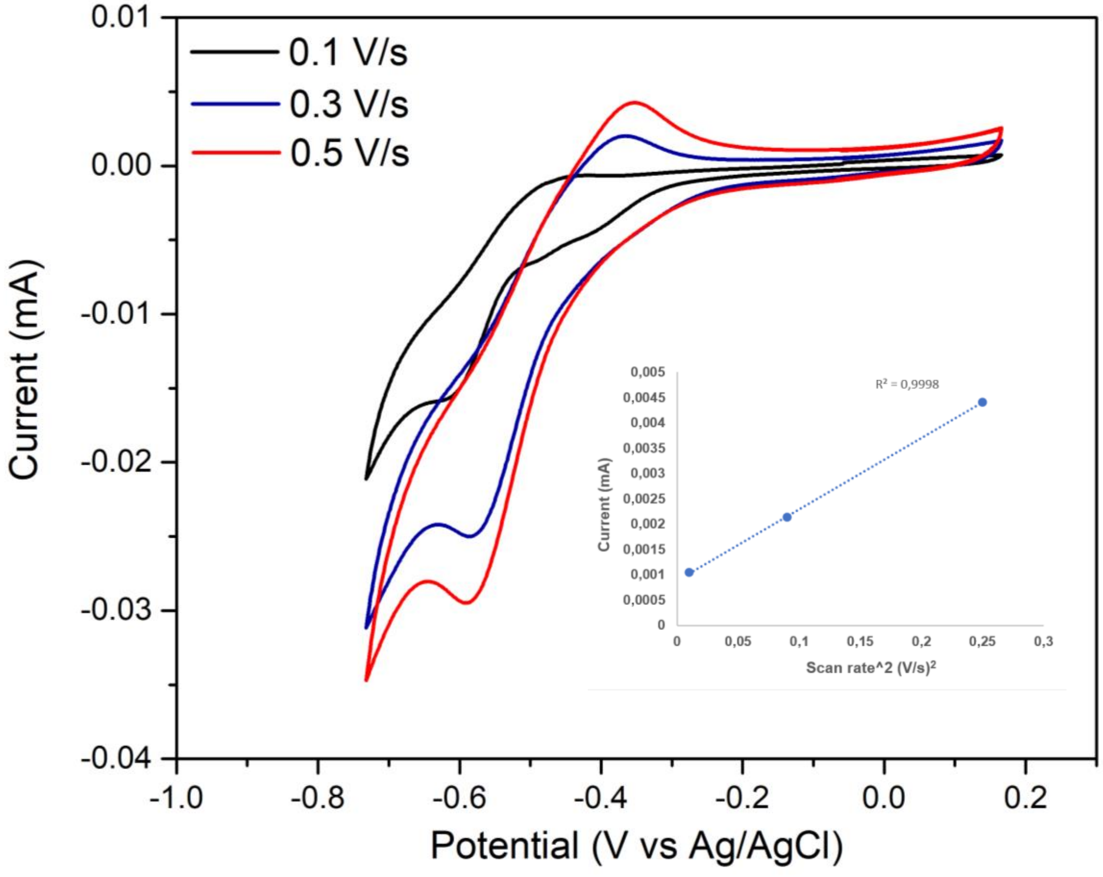

Figure S25. Solid-state CV of **PTMTC$^{R@NR}$-Zn MORF** crystals in CH_2Cl_2 using $TBAPF_6$ 0.1 M as electrolyte and different scan rate. Platinum wire was used as the counter electrode and silver wire as the pseudoreference electrode. Ferrocene was added as internal standard. All potentials are reported versus Ag/AgCl. The inset shows the linear relationship of cathodic peak current vs. the square root of the scan rate.

6. Crystal structure of PTMTC^{NR}-Zn MOF and PTMTC$^{R@NR}$-Zn MORF

One single-crystal with block shape of **PTMTC^{NR}-Zn MOF** or of **PTMTC$^{R@NR}$-Zn MORF** was manually selected from the crystallization vial, and was mounted on a glass fiber with the help of silicon grease. The crystal was rapidly covered with silicon grease in order to prevent the solvent to evaporate; failure to do so resulted in a complete loss of crystallinity (with other crystals, that were therefore discarded). Data were collected at 180(2) K on a Bruker X8 Kappa APEX II charge-coupled device (CCD) area-detector diffractometer (Mo Ka graphite-monochromated radiation, λ = 0.71073 Å) controlled by the APEX2 software package,[1] and equipped with an Oxford Cryosystems Series 700 cryostream monitored remotely using the software interface Cryopad.[2] Images were processed using the software package SAINT+,[3] and data were corrected for absorption by the multi-scan semi-empirical method implemented in SADABS.[4] The structure was solved using the direct methods algorithm implemented in SHELXS-97,[5,6] which allowed the immediate location of the majority of the atoms. All remaining non-hydrogen atoms were located from difference Fourier maps calculated from successive full-matrix least squares refinement cycles on F^2 using SHELXL-97.[5,7] All non-hydrogen atoms were successfully refined using anisotropic displacement parameters.

Hydrogen atoms bound to carbon were located at their idealized positions using appropriate HFIX instructions in SHELXL (43 for the aromatic and vinylic, 23 for the $–CH_2–$ moieties and 13 for the chiral tertiary carbon atoms) and included in subsequent refinement cycles in riding-motion approximation with isotropic thermal displacements parameters (Uiso) fixed at 1.2 times Ueq of the atom to which they are attached.

Crystallographic data for the structures reported in this paper have been deposited with the Cambridge Crystallographic Data Centre as supplementary publication Nº. CCDC 2266389-2266390. Copies of the data can be obtained free of charge on application to CCDC, 12 Union Road, Cambridge CB2 2EZ, U.K. Fax: (+44) 1223 336033. E-mail: deposit@ccdc.cam.ac.uk.

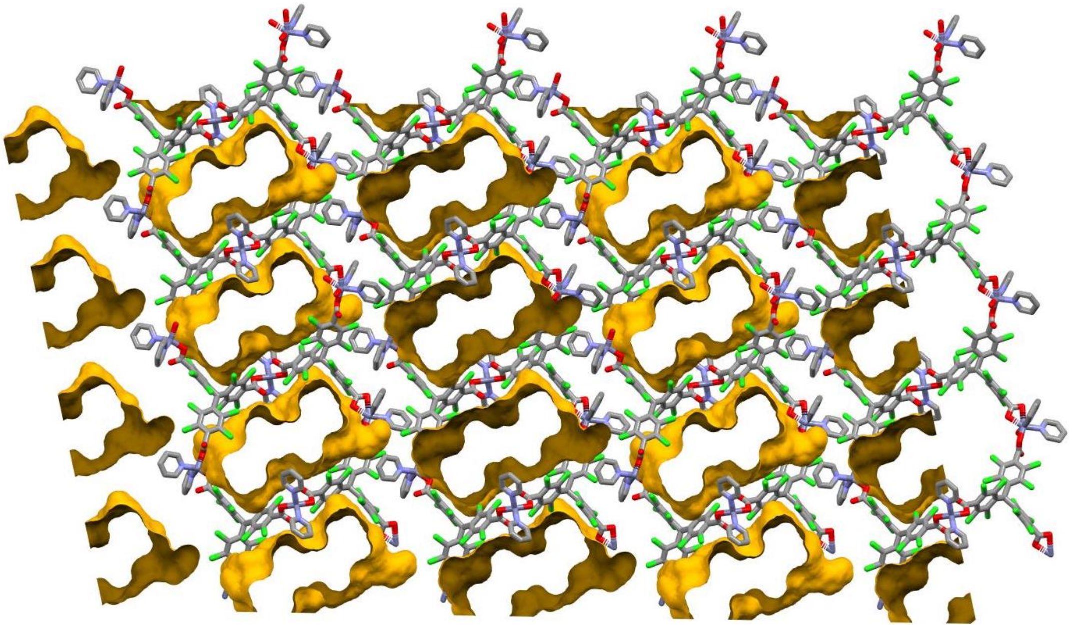

Figure S26. Partial view of the crystal structure of **PTMTC^{NR}-Zn MOF** showing the solvent-accessible surface (~37 %).

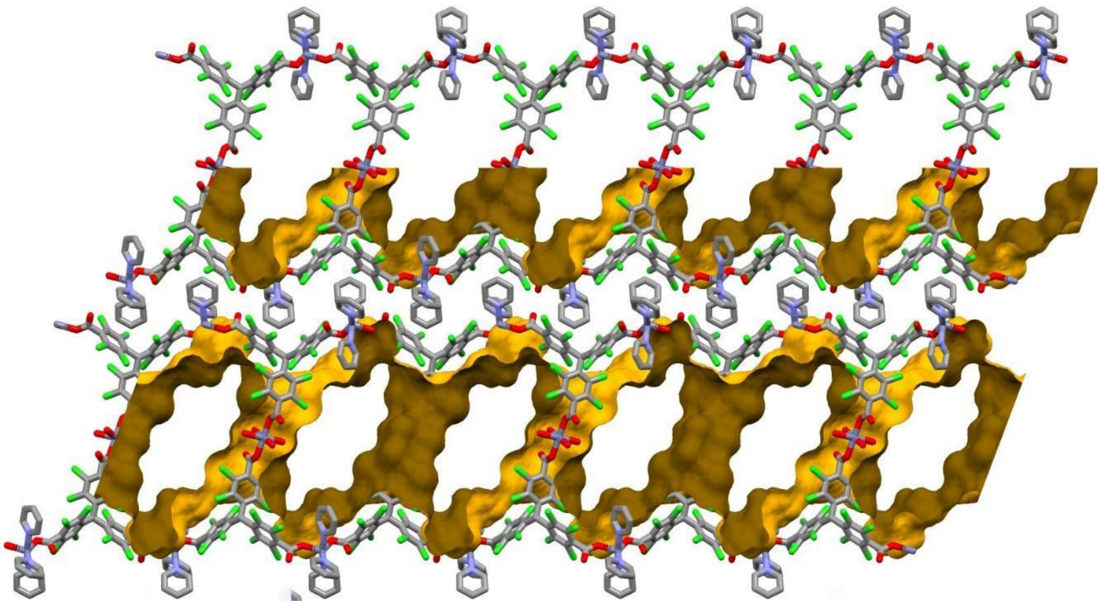

Figure S27. Partial view of the crystal structure of **PTMTC$^{R@NR}$-Zn MORF** showing the solvent-accessible surface (~29 %).

7. Kelvin probe force microscopy (KPFM)

KPFM experiments were done at rarefied atmosphere (air pressure below 5×10^{-1} Torr) and room temperature (about 25°C) using Park NX HiVac microscope (Park Systems). A supersharp AFM probe SSS-NCHR (Nanosensors, Switzerland) with the tip curvature radius below 2 nm was used. The first resonance frequency of the cantilever (298 kHz) was used for the non-contact feedback with the set point fixed at 90% of the amplitude of free vibrations (7 nm) that corresponds to a tip-sample distance about 18 nm. A single-pass KPFM mode was implemented using AC voltage of amplitude 0.1 V and frequency of 17 kHz. No DC voltage bias was applied between the tip and the sample. The registered KPFM signal was analyzed by a built-in lock-in amplifier with time constant 30 ms and sensitivity 0.1 V. Scan size was set 25×25 nm with 256×256 points. In total, 18 scans at different places of one **PTMTC^{NR}-Zn MOF** and two **PTMTC$^{R@NR}$-Zn MORF** crystals were measured. For each scan line, forward and backward KPFM signals were collected and analyzed jointly to insure the reproducibility of the measurements. However, raw scans contain much amount of noise. Therefore, the following pre-treatment scheme was performed using Gwyddion software. First, high frequency noise of electrical origin was removed from the scans using FFT filtration. The instability of the mechanical system especially prominent at small size scans leads to the mismatch even between consequent scans. Therefore, the cross-correlation analysis between forward and backward scans was applied to reveal the regions with high correlation (correlation score higher than 70% of the maximum). The obtained mask was applied to the FFT filtered scans and the average and median values of the KPFM potential were extracted for each region. Such pre-treatment was applied for all pairs of forward and backward scans measured for each crystal. Then, the results were combined together and plotted as histograms. Such analysis allows separating the reproducible signals only thus related to the effect of the sample. The sample's work function, φ_s, depends on the work function of the tip, φ_t, and the KPFM amplitude, V_{KPFM}: $\varphi_s = \varphi_t - eV_{KPFM}$, where e – is an elementary charge. Therefore, for the same tip, the measured KPFM potential signal is proportional to the sample's work function: $V_{KPFM} = (\varphi_t\text{-}\varphi_s)/e$.

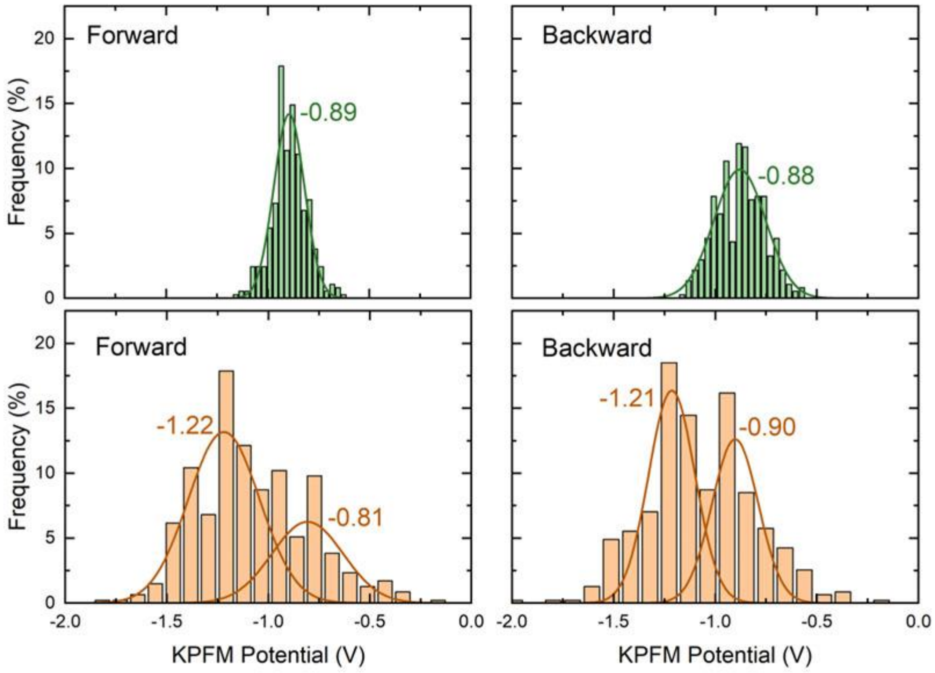

Figure S28. The distributions of KPFM potential measured over the surface of **PTMTC^{NR}-Zn MOF** (green) and **PTMTC$^{R@NR}$-Zn MORF** (red) crystals for different scanning directions. Solid curves show the histogram fitting with Gauss function.

8. Breathing behavior and optical proeprties of PTMTC$^{R@NR}$-Zn MOF

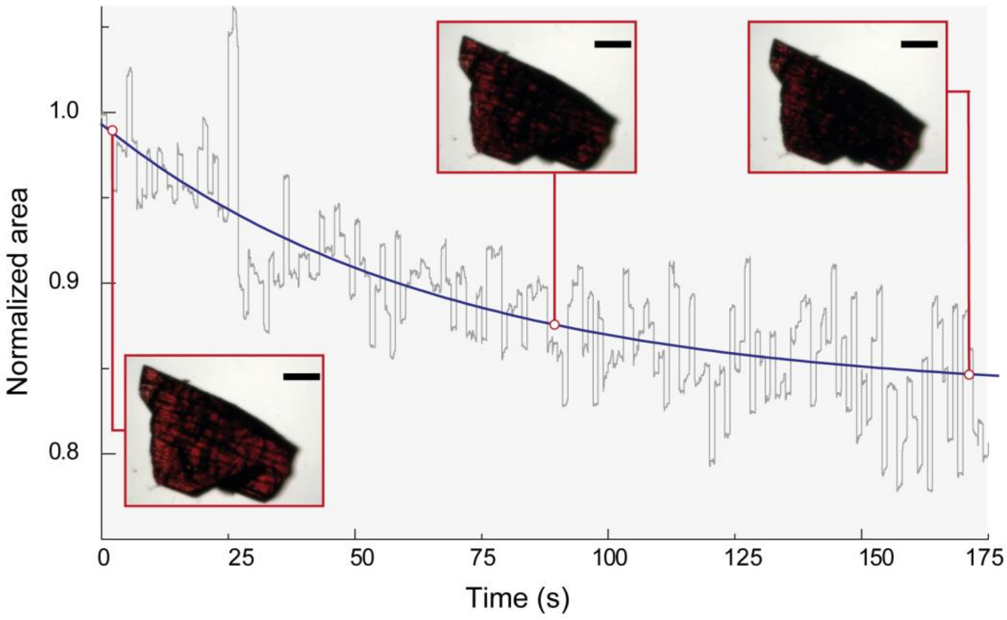

Figure S29. Time dependence of the normalized area calculated from the images of a **PTMTC$^{R@NR}$-Zn MORF** crystal taken with an optical microscope at different times. The crystal was initially in contact with ethanol (0 s) that in the end completely evaporated (180 s) reducing its area by about 15 %. Scalebars correspond to 0.5 mm.

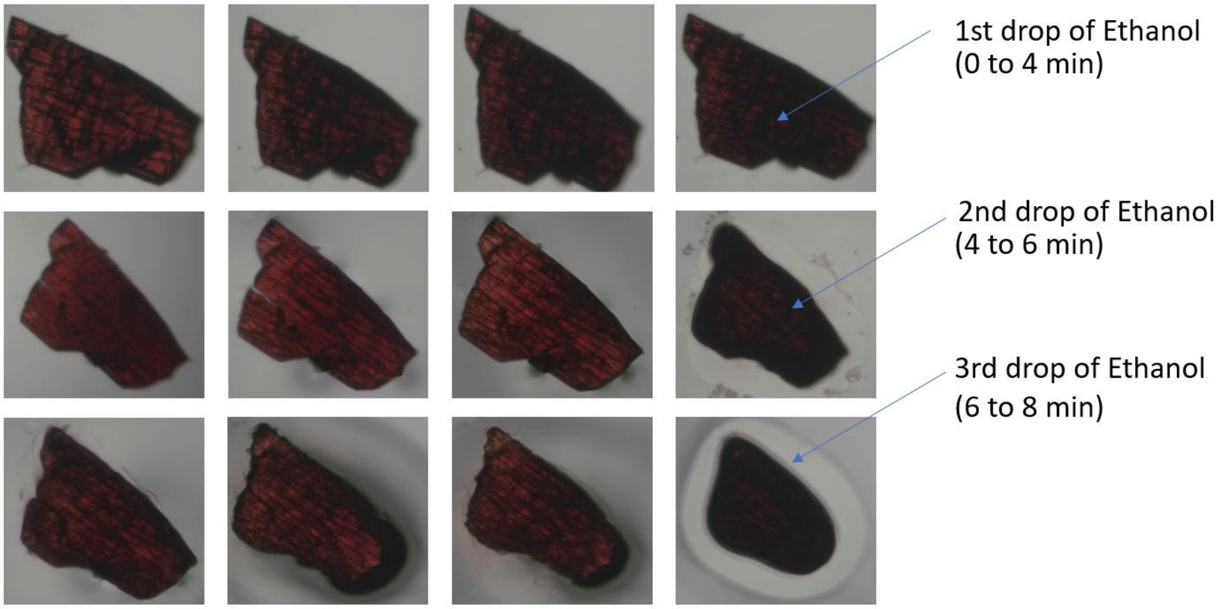

Figure S30. Optical images of **PTMTC$^{R@NR}$-Zn MORF** crystal after being exposed to one drop of ethanol at different times.

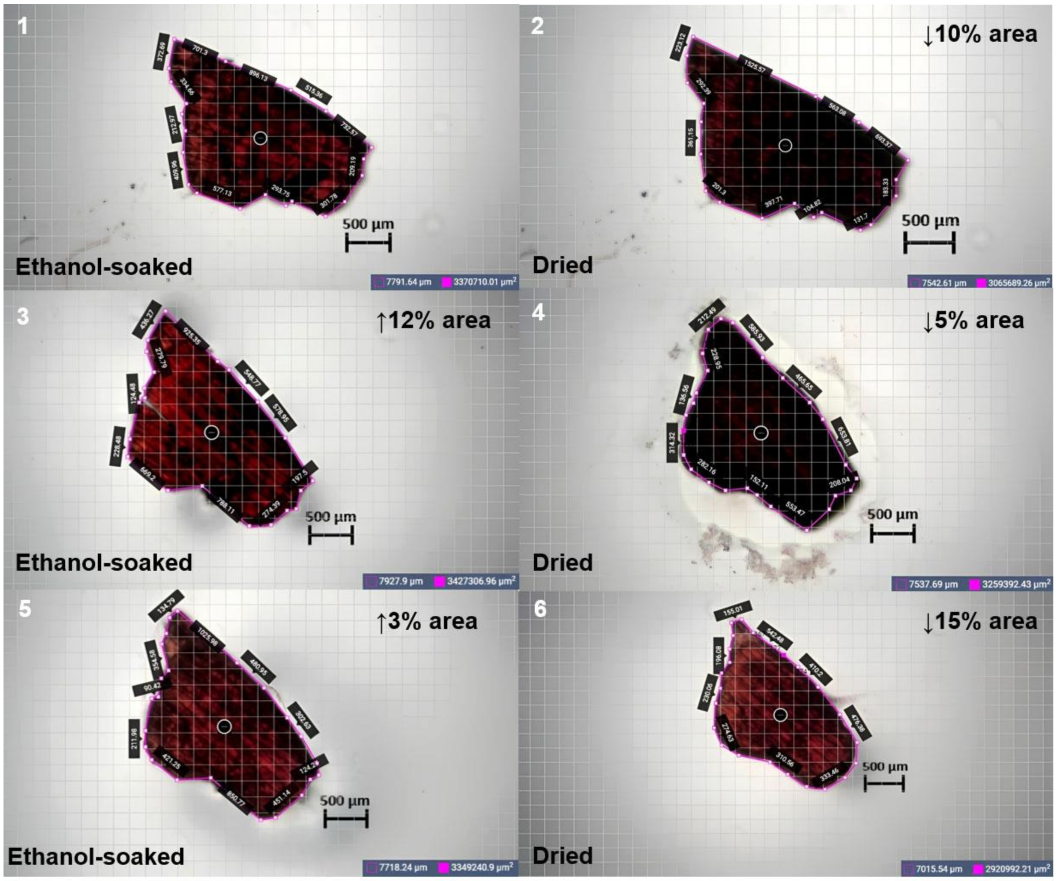

Figure S31. Calculated areas for ethanol-soaked and dried crystals of **PTMTC$^{R@NR}$-Zn MORF** after three solvation-evaporation cycles using the same crystal.

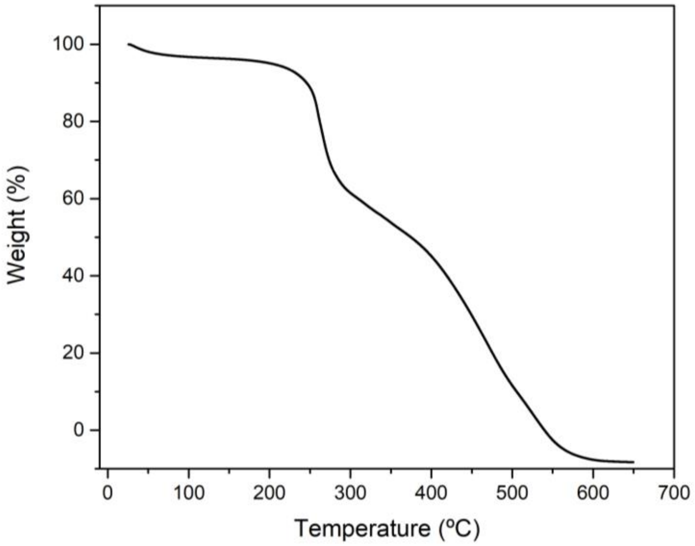

Figure S32. Thermogravimetric analysis (TGA) profile of **PTMTC$^{R@NR}$-Zn MORF** at a heating rate of 5ºC/min.

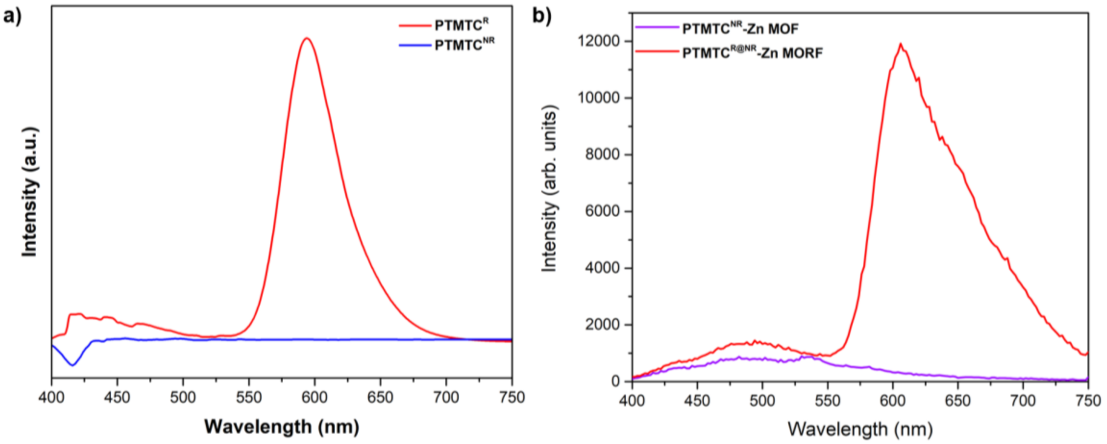

Figure S33. a) Emission spectra of **PTMTC^{R} and PTMTC^{NR}** in $CHCl_3$ (0.05 mM) upon 377 nm excitation at room temperature. b) Emission spectra upon 377 nm excitation of a single crystal of **PTMTC^{NR}-Zn MOF** and **PTMTC$^{R@NR}$-Zn MORF** at room temperature.

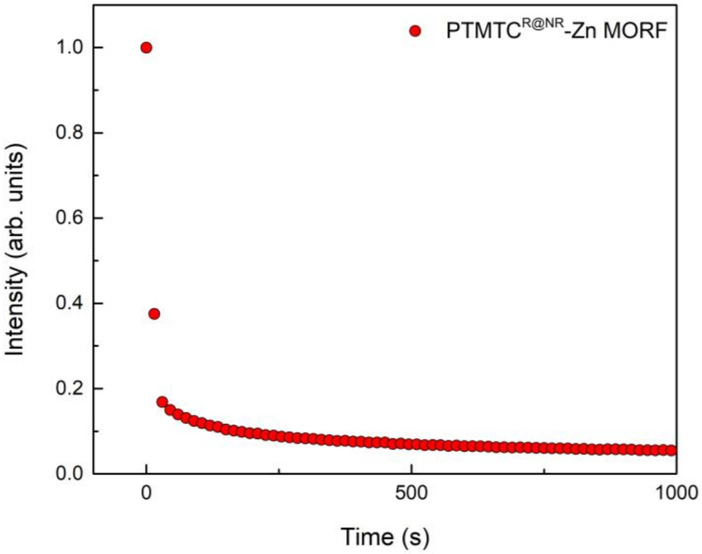

Figure S34. Time evolution of the emission intensity of **PTMTC$^{R@NR}$-Zn MORF** single crystal over irradiation time by 378 nm excitation.

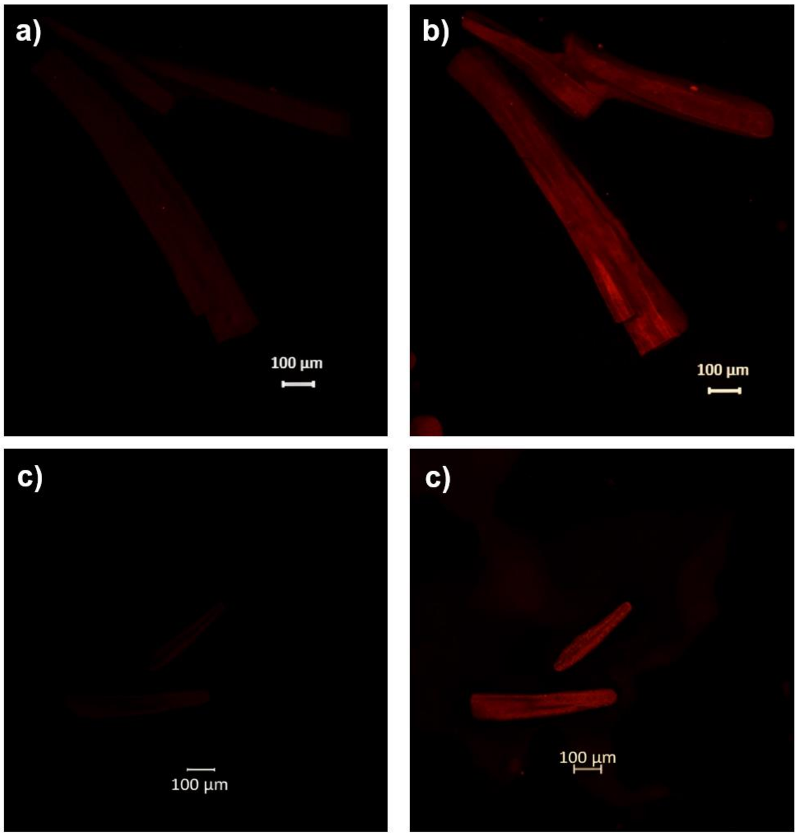

Figure S35. Confocal microscope images of (a,c) dried and ethanol-soaked (b) and propanol-soaked (d) crystals of **PTMTC$^{R@NR}$-Zn MORF** illuminated with 400 nm light at room temperature.

9. References

[1] APEX2, Data Collection Software Version 2.1-RC13, Bruker AXS, Delft, The Netherlands 2006.

[2] Cryopad, Remote monitoring and control, Version 1.451, Oxford Cryosystems, Oxford, United Kingdom 2006.

[3] SAINT+, Data Integration Engine v. 7.23a © 1997-2005, Bruker AXS, Madison, Wisconsin, USA.

[4] G. M. Sheldrick, SADABS v.2.01, Bruker/Siemens Area Detector Absorption Correction Program 1998, Bruker AXS, Madison, Wisconsin, USA.

[5] G. M. Sheldrick, Acta Cryst. A, 2008, 64, 112-122.

[6] G. M. Sheldrick, SHELXS-97, Program for Crystal Structure Solution, University of Göttingen 1997.

[7] G. M. Sheldrick, SHELXL-97, Program for Crystal Structure Refinement, University of Göttingen 1997.
